# Long-tailed class I myosins rely on tail-mediated phosphoinositide recognition for specific membrane recruitment

**DOI:** 10.1186/s12964-025-02528-x

**Published:** 2025-12-04

**Authors:** Girish Rajendraprasad, Despoina Kyriazi, Peter Franz, Almke Bader, Muriel Erent, Petra Uta, Matthias Preller, Tim Scholz, Georgios Tsiavaliaris

**Affiliations:** 1 Cellular Biophysics, Institute for Biophysical Chemistry, Hannover Medical School, Carl-Neuberg-Str. 1, Hannover, 30625 Germany; 2 Biomedical Sciences, Warwick Medical School, The University of Warwick, Coventry, CV4 7AL UK; 3 Department of Molecular and Cell Physiology, Hannover Medical School, Carl-Neuberg-Str. 1, Hannover, 30625 Germany; 4 Bonn-Rhein-Sieg University of Applied Sciences, Grantham-Allee 20, Rheinbach, 53757 Germany

**Keywords:** Myosin-1, Myosin-1B, Myosin-1C, Myosin-1D, Actin, Microtubules, Phosphatidylinositol, Actin, Endocytosis, Macropinocytosis, Phagocytosis, Mitosis, Dictyostelium discoideum

## Abstract

**Background:**

Class I myosins are essential mediators of membrane–cytoskeleton interactions that support key cellular processes such as endocytosis, secretion, intracellular trafficking, and mitosis. However, the mechanisms driving isoform-specific targeting to membrane domains enriched in signaling lipids as well as their stage-dependent recruitment to mitotic structures during cell division remain poorly defined.

**Methods and approach:**

Using *Dictyostelium discoideum* as a highly phagocytic cell model, we demonstrate that long-tailed myosin-1 isoforms (myosin-1B, -1C, and − 1D) exhibit distinct lipid and cytoskeletal binding profiles shaped by their modular tails and variations within the phosphoinositide binding motif. Homology-based structural modelling of the PH-like lipid binding domain within the TH1 sequence, combined with molecular docking explains their differential lipid affinities. Kinetic equilibrium modelling with quantitative data suggests these differences enable cooperative or competitive isoform localization within cells providing a mechanism for temporally controlled recruitment of the myosins in response to dynamic changes in membrane composition and expression profiles. These biochemical insights are corroborated by confocal live-cell imaging, which reveals phosphoinositides-dependent localization dynamics and isoform-specific targeting of the myosins during vegetative growth and mitotic progression.

**Results:**

Myosin-C exhibits phosphoinositide binding preferences nearly reciprocal to those of myosin-1D, especially between mono- and triple phosphorylated phosphoinositides, and shows the strongest tail-mediated, ATP-independent actin binding. Myosin-1B, in contrast, displays low affinity for monophosphorylated phosphoinositides, intermediate actin binding ability, and no microtubule interaction. The comparable affinities of all three myosins for PI(3,5)P₂ and PI(4,5)P₂, the major PIP species at the cell cortex, facilitate their accumulation at membrane protrusions. Live-cell imaging confirms that myosin-1D preferentially associates with PI(3,4,5)P₃- and PI(3)P-enriched endosomes during macropinocytosis and phagocytosis, consistent with its higher binding affinity for these phosphoinositides. Conversely, myosin-1C localization is governed by both actin and phosphoinositides, enabling a rapid dissociation from early endosomes to retarget the cortex and accumulate at actin-rich phagocytic cup tips. Upon mitotic entry, myosin-1D, similar to myosin-1C, redistributes from endosomal compartments to the mitotic apparatus, where it decorates membrane-enclosed nuclear chromatin masses through its TH1 domain and later associates with spindle pole microtubules. This contrasts with myosin-1C, which selectively targets spindle microtubules throughout mitosis, reflecting its stronger microtubule-binding affinity. Inhibition of PI3-kinase disrupts membrane recruitment of both isoforms, confirming their phosphoinositide-dependent localization. These findings reveal an isoform-specific mechanism underlying myosin-1 targeting during endocytosis and mitosis.

**Conclusion:**

Collectively, these findings establish a phosphoinositide- and cytoskeleton-guided mechanism that governs myosin-1 isoform-specific functions, providing new insights into how motor proteins interpret complex lipid and cytoskeletal cues to regulate membrane remodelling and cytoskeletal dynamics across cellular states.

**Supplementary Information:**

The online version contains supplementary material available at 10.1186/s12964-025-02528-x.

## Background

Directional cell migration, cell shape changes, and membrane remodeling during endocytosis and secretory events depend on the precise coordination of actin cytoskeleton dynamics with membrane organization [1, 2]. Class I myosins, monomeric, ATP-dependent motors, play key roles in these processes [3]. By binding both actin filaments and phospholipids, they function at the actin-membrane interface as motorized cross-linkers, generating local forces that couple cortical actin remodeling to membrane deformation [4]. Through these activities, the myosins drive vesicle trafficking, coordinate cortical tension, and assist in endocytosis, adhesion dynamics, and cellular motility [5–10]. The observed functional diversity among class I myosins arises from differences in the kinetic properties of their N-terminal motor domains [11–16] as well as structural variations within the tail domains [17–22].

All class I myosins share a conserved tail homology 1 (TH1) domain exhibiting pleckstrin homology (PH)-like lipid-binding properties. Long-tailed isoforms extend this architecture with a glycine-rich tail homology 2 (TH2) region and a Src homology 3 (SH3) domain [3], which mediate interactions with scaffolding proteins and signaling complexes, together orchestrating membrane associations, cargo recognition, and recruitment of actin-regulatory factors [23–25]. Particularly, the membrane association predominantly relies on electrostatic interactions between basic residues in the TH1 domain and negatively charged phosphoinositides, including PI(3,4)P₂ PI(4,5)P₂, and PI(3,4,5)P₃, whose spatial and temporal distribution is dynamically regulated by PI3K, PLC, and phosphatase signaling [26]. While PI(4,5)P₂-dependent recruitment has been demonstrated for multiple myosin I isoforms [17, 18, 21], broader phosphoinositide-binding specificity, encompassing also mono- and triple phosphorylated PIPs has also been reported [27–29].

The presence of multiple class I myosin isoforms within a single cell—ranging from two in yeast to eight in mammals—implies both overlapping and specialized functions. In mammals, the two long-tailed isoforms, myosin-1e and myosin-1f, localize to PI(3,4,5)P₃-enriched membrane domains and mediate cell type-specific roles in podocyte architecture, membrane stiffness, immune cell migration, and phagocytosis [10, 30, 31]. Notably, mutations in myosin-1e have been associated with focal segmental glomerulosclerosis [32], while heterozygous missense mutations in the short-tailed myosin-1c, which is critical for mechanotransduction in the inner ear, are associated with bilateral sensorineural hearing loss [33], and myosin-1 isoforms in *Drosophila* are essential for the establishment of left-right asymmetry [34], highlighting the physiological importance of isoform-specific cellular activities of the motors. Despite their functional divergence, myosin-1 isoforms across species share a conserved core ability to coordinate actin remodeling with membrane dynamics, processes critical for regulation of cell behavior. *Dictyostelium discoideum* was among the first model organisms to demonstrate the critical involvement of class I myosins in endocytic processes, cortical tension, and cell migration [35]. Although some functional redundancy exists among myosin-1s across species [36–38], genetic ablation studies demonstrate that combinatorial loss of multiple isoforms results in more severe phenotypes, including perturbed membrane trafficking and compromised cytoskeletal integrity [38].

Beyond lipid binding, class I myosins serve as scaffolds for actin-regulatory machinery directly engaging actin or recruit effectors such as WASP and the Arp2/3 complex to sites of membrane remodeling, driving localized actin assembly essential for vesicle internalization and trafficking [39–43]. Post-translational modifications, including phosphorylation downstream of PI3K and PLC, fine-tune myosin-1 membrane affinity and motor activity in response to extracellular cues [44]. Additionally, SH3 domain-mediated interactions integrate class I myosins into broader signaling and cytoskeletal networks, ensuring spatial coordination of force production with membrane architecture [43, 45]. Moreover, emerging evidence reveals nuclear functions for class I myosins as well [45–47]. Nuclear myosin I (NMI) referred to a myosin-1c associates with RNA polymerases I and II and chromatin remodeling complexes to regulate transcription and chromatin accessibility [48]. In *Xenopus* and *Dictyostelium*, specific myosin-1 isoforms localize to the spindle apparatus, linking F-actin and microtubules (MT) to ensure spindle stability and chromosome segregation [49, 50]. These findings highlight an evolutionary conservation with shared and versatile functions of class I myosins, whose isoform-specific motor kinetics and tail architectures dictate subcellular localization and function.

While motor domain kinetics are well established as key determinants regulating the duty ratio of the ATPase cycle to facilitate tension generation, cargo transport, or rapid force production, the contributions of tail domains remain less well understood [13, 51]. Despite extensive investigation, the mechanisms underlying myosin-1 membrane targeting and the molecular determinants that confer isoform-specific functions within broader cytoskeletal networks remain poorly defined [52].

To extend our understanding of the mechanisms governing class I myosin recruitment at membranes, we employed the professional phagocyte *Dictyostelium discoideum*, which expresses seven class I myosin isoforms—three long-tailed (myosin-1B, myosin-1C, myosin-1D) and four short-tailed (myosin-1 A, myosin-1E, myosin-1 F, myosin-1 K) [53]. Focusing on the long-tailed isoforms, and particularly on myosin-1C and myosin-1D, we conducted comparative in vitro and in vivo analyses investigating tail-mediated mechanisms governing membrane-cytoskeletal crosstalk during endocytosis, additionally revealing nuclear association during mitosis.

## Methods

### Plasmid construction

Plasmids encoding YFP-fusions of full-length myosin-1B (NCBI: XM_631290.1), myosin-1C (NCBI: XM_637968.1), and myosin-1D (NCBI: XM_638354.1) were used as templates [15, 50, 54] for the generation of expression vectors encoding GST-and YFP-tagged tail constructs. The myosin-1B tail (amino acids 717–1111) and myosin-1D tail (amino acids 717–1113) constructs were amplified by PCR using primers containing BamHI and BsrGI sites, then cloned into pGEX-6P2 (GE Healthcare) and pGEX-6P2–eYFP–mcs vectors to introduce N-terminal GST and C-terminal YFP tags, respectively. The myosin-1D tail-TH1 construct (aa 721–913), comprising only the TH1 domain, was generated from [plasmid/source] by BamHI/PvuII digestion and cloned into BamHI/SmaI-cut pGEX-6P2. The myosin-1D-tail-ΔSH3 construct, lacking the SH3 domain (aa 969–1016), was generated by PCR-mediated deletion from pDXA-YFP-myosin-1D, and the fragments were ligated to obtain pDXA-YFP-myosin-1D-ΔSH3. For the bacterial expression of GST-tagged tail version of myosin-1D-ΔSH3, pDXA-YFP-myosin-1D-ΔSH3 was cut with restriction enzymes BamHI and XhoI and inserted into pGEX-6P2 using the same restriction sites. All constructs were sequence-verified. The RFP-myosin-1C plasmid was generated by subcloning the XhoI/XbaI fragment from YFP-mcs-myosin-1C into the pDXA–YFP–mcs vector.

### Cell lines and protein preparation

*Dictyostelium discoideum* AX2 cells were used throughout the experiments, including confocal microscopy and live-cell imaging experiments. Transformation with the respective plasmids and cultivation followed the protocols described [15]. Cells expressing both RFP-myosin-1C and YFP-myosin-1D were generated by transforming YFP-myosin-1D-expressing cells with the RFP-myosin-1C plasmid, using hygromycin B resistance for selection. Transformants were maintained in HL-5c medium at 21 °C and selected with 10 µg/ml G418. Double transformants were selected using 10 µg/ml G418 and 30 µg/ml hygromycin B. Synchronization experiments followed established protocols [55, 56]. Tail constructs were recombinantly expressed and purified from *E. coli* Rosetta pLys-S cells (Merck, Darmstadt) as described [50]. Cells were grown in LB medium at 30 °C to an OD_595_ of 0.6–0.8, then induced with 0.5 mM IPTG. Following harvest by centrifugation (4000 g), cells were lysed in buffer containing 50 mM Tris-HCl (pH 8.0), 300 mM NaCl, 1 mM MgCl_2_, 1 mM EDTA, 5 mM benzamidine, 5 mM DTT, 4 mM PMSF, 0.5 mg/ml lysozyme, four pellets complete protease inhibitor cocktail (Roche), 1000 U benzonase (Merck), and 1% Triton X-100. Lysates were clarified by centrifugation (20,000 g), and supernatants were applied to glutathione-Sepharose columns. Bound proteins were washed with 10 column volumes of buffer A (50 mM Tris-HCl, pH 7.5, 300 mM NaCl, 1 mM EDTA, 2 mM DTT, 2 mM benzamidine) and eluted using a linear gradient from buffer A to buffer B (buffer A supplemented with 10 mM reduced glutathione). Proteins were further purified by size exclusion chromatography (HiLoad 26/60 Superdex 200 pg, GE Healthcare). Concentrated proteins were dialyzed into storage buffer (25 mM Tris-HCl, pH 7.5, 50 mM arginine, 50 mM glutamate, 300 mM NaCl, 2 mM DTT, 3% sucrose), snap-frozen in liquid nitrogen, and stored at − 80 °C.

### Cosedimentation experiments

Phospholipid binding assays were performed using commercially available lipid vesicles (PolyPIPosomes™, 200 nm diameter; Echelon Biosciences) composed of 95% PE + PC and 5% of the phosphoinositide of interest. Accessible phospholipid concentration was calculated as half of the total phospholipid content. Myosin-1 tail (500 nM) in co-sedimentation buffer (10 mM Tris, pH 7.5, 100 mM NaCl, 1 mM EGTA) was incubated with increasing concentrations of PolyPIPosomes™ (accessible PIP range: 10 nM to 10 mM) at room temperature for 30 min, followed by centrifugation at 25,000 g to separate bound and unbound fractions. Bound myosin-1 tail was quantified either by measuring fluorescence intensity of the supernatant (Jasco FP-6500 fluorimeter, excitation 514 nm) or by western blot of pellet and supernatant fractions using anti-GST antibody as indicated in the experiments. Myosin-1 tail binding to F-actin and MT was analyzed by cosedimentation assays detecting YFP fluorescence. F-actin binding assays used buffer containing 5 mM Tris (pH 7.4), 100 mM KCl, 0.04% NaN_3, and 2 mM MgCl_2_. Microtubule binding assays employed 100 mM PIPES (pH 6.8), 2 mM MgCl_2_, 1 mM EGTA, 20 µM paclitaxel, and 1 mM GTP. Samples were centrifuged at 20,000 g for 30 min at 4 °C (F-actin) or 30 °C (microtubules). Fluorescence intensities of pellet and supernatant fractions were quantified as described above.

### Fluorescence microscopy

TIRF microscopy of myosin-1 binding to F-actin and microtubules was conducted as previously described [50]. Microtubule depolymerization rates were quantified by measuring individual microtubule length reduction over time using Andor Solis software. Cellular localization of fluorescently tagged myosins and time-lapse imaging were performed on an inverted Leica TCS SP2 AOBS confocal microscope and a Leica DMi8 M system equipped with a 63×/1.4 NA oil immersion objective. Bright-field fluorescence microscopy employed an Olympus IX81 inverted microscope with a 60×/1.49 NA oil immersion lens (ApoN, Olympus). For imaging, cells were seeded on glass-bottom dishes (Ibidi), washed twice with MES buffer (20 mM MES, pH 6.8, 0.2 mM CaCl₂, 2 mM MgCl₂), and maintained in this buffer during acquisition. Phagocytic uptake assays were performed following a 2-hour starvation, after which heat-killed yeast particles were added and time-lapse images recorded. Immunofluorescence fixation followed established protocols [50, 57]. To visualize myosin-1 tail binding to the nuclear membrane, nuclei were isolated from *Dictyostelium* cells [58], stained overnight with DAPI (1:10,000), then incubated with 1 µM myosin-1 tail construct for 30 min. Nuclei were placed in glass-bottom dishes for settling and imaged by confocal microscopy with 0.5 μm Z-stack slices. Image processing was performed using Leica Confocal Software, and quantitative analysis was conducted with ImageJ (NIH, Bethesda, MD, USA). Fluorescence intensity profiles were obtained by drawing lines along the regions of the cups, as illustrated in Fig. 5. Fluorescence data were normalized to the maximum intensity and interpolated using the interpolation algorithm in Origin to account for variations in cup length. Frames showing association of myosin-1C or myosin-1D with the cups were quantified. Colocalization analysis was conducted using the JaCOP plugin in Fiji, applying the Otsu threshold method. Normality of the data was assessed with the Shapiro-Wilk test. If both datasets passed the normality test, statistical significance was evaluated using an unpaired t-test; otherwise, a Mann-Whitney test was applied. The p values, as well as the statistical tests used are indicated in the corresponding figure legends. Data are presented as averages ± standard deviation (S.D.).

### Structural modelling and kinetic modelling of binding equilibria

Models of the PH domains of *Dictyostelium discoideum* myosin-1B, −1C, and − 1D were generated using MODELLER [59], based on the crystal structure of human myosin-1C (PDB: 4Z8G). Model selection relied on the MODELLER objective function and discrete optimized protein energy (DOPE) score. The position of PI(3,4,5)P₃ in the binding pocket was derived from the co-crystal structure of PDK1 bound to PI(3,4,5)P₃ (PDB: 1W1D). Structural models underwent geometry optimization in Schrödinger MacroModel (version 11.2) using the OPLS3 force field, applying spatial position constraints to the protein backbone atoms and ligand. For myosin-1C, atoms in the β1–β2 loop were unconstrained to allow refinement, as this loop initially occluded the PI(3,4,5)P₃ binding site. Prior to energy minimization, models were prepared using the Schrödinger Protein Preparation Wizard ((Schrödinger Release 2016-2: Schrödinger Suite 2016-2 Protein Preparation Wizard; Epik version 3.6, Schrödinger, LLC, New York, NY, 2016; Impact version 7.1, Schrödinger, LLC, New York, NY, 2016; Prime version 4.4, Schrödinger, LLC, New York, NY, 2016). Myosin–PI(3,4,5)P₃ interactions were analyzed in Schrödinger Maestro (version 10.6). Molecular docking was performed with AutoDock4 using the Lamarckian Genetic Algorithm. Protein and ligand structures were prepared using AutoDockTools [60]. Kinetic modeling of binding equilibria was performed using the software Kintek Explorer (Version 8.0 with FitSpace Explorer™ and SpectraFit™).

## Results

### Long-tailed myosin-1s display distinct phosphoinositide binding properties

Membrane association of class I myosins is predominantly mediated by the basic tail homology 1 (TH1) domain [23], which adopts a pleckstrin homology (PH)-like fold despite lacking a canonical PH motif [61]. Sequence alignments reveal notable isoform-specific variations of residues implicated in coordinating phosphorylated phosphoinositides (PIPs), differing in both number and electrostatic properties, as well as sequence context (Supplementary fig. S1), indicating the potential for distinct phospholipid interactions. To investigate PIP specificity, we initially performed lipid overlay assays using nitrocellulose membranes spotted with various phosphoinositides (Supplementary fig. S2a). While the positive control dynamin A exhibited the expected lipid-binding specificity [62], the three *Dictyostelium* myosin tails demonstrated broad binding across the tested lipids including phosphorylated and unphosphorylated inositides without clear selectivity or preference for a particular species. Recognizing the limitations of the assay, which does not account for critical parameters like membrane curvature, lipid packing, lipid accessibility, and lateral mobility that can critically influence binding, we established a quantitative, fluorescence-based sedimentation assay with artificial unilamellar liposomes of defined size and lipid composition, comprising phosphatidylethanolamine (PE) and phosphatidylcholine (PC) as the dominant species and single phosphoinositides present in physiological concentrations (Supplementary fig. S2b). To facilitate sensitive detection of the bound fraction, recombinant tail constructs were equipped with YFP and/or GST (Supplementary fig. S3). Since both, fluorescence and immunoblot-based detection yielded comparable results as exemplified for myosin-1D, providing similar affinity constants for PIP2 (Supplementary fig. S2b), we continued the analysis using the fluorescence-based assay due to its throughput advantage and superior sensitivity.

**Fig. 1:**
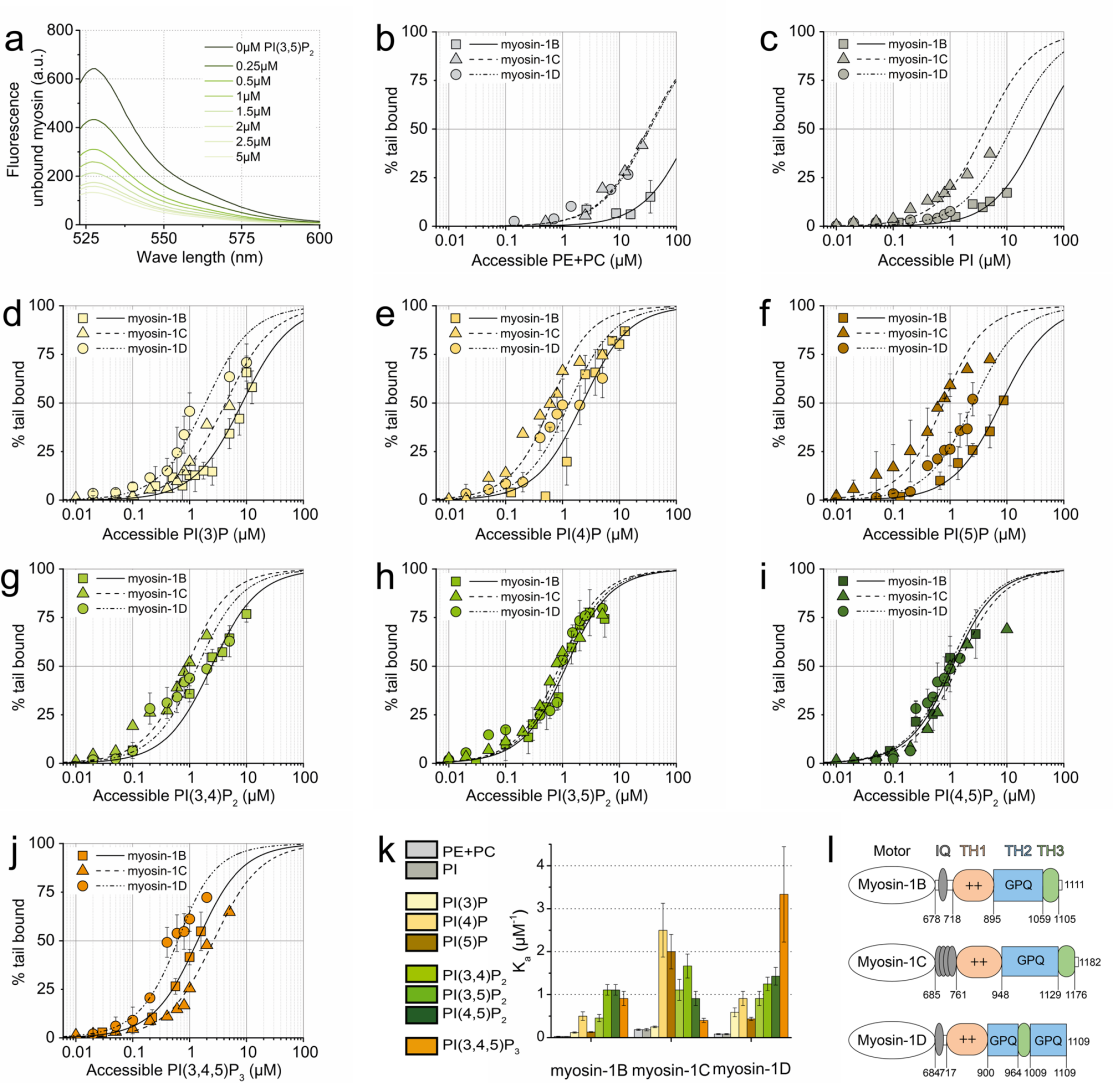
Myosin-1 tail binding to phosphoinositides. **a** Representative fluorescence spectra of supernatants from co-sedimentation assays with YFP-tagged myosin-1D tail and increasing concentrations of lipid vesicles containing PI(3,5)P₂. The decrease in fluorescence intensity with rising phosphoinositide concentration reflects the fraction of myosin-1D tail bound to vesicles. **b–j** Half-logarithmic plots depicting the fraction of myosin-1 tail bound to lipid vesicles as a function of accessible phospholipid concentration. Data represent mean ± SD from two to four independent experiments. Lines show best fits to the quadratic binding equation: *[tail•PIP*_*x*_*]*_*eq*_ *= 1/2∙([tail]+[PIP*_*x*_*] + K*_*d*_*)-√([tail]+[PIP*_*x*_*] + K*_*d*_*)*^*2*^*−4∙[tail]∙[PIP*_*x*_*])/[tail]*, where K_d_ is the dissociation constant and brackets denote concentrations of interacting species. **k** Bar graph summarizing equilibrium association constants (K_a_ = 1/K_d_) derived from fits. Error bars indicate standard error of the fits. **l** Domain architecture of the *Dictyostelium* long-tailed class-1 myosins

Titration experiments delineated both shared and isoform-specific phosphoinositide-binding affinities among the myosins, with dissociation constants ranging from submicromolar to micromolar concentrations (Fig. 1; Table 1). All constructs exhibited negligible binding to liposomes devoid of phosphoinositides (Fig. 1b) or containing unphosphorylated phosphatidylinositol (Fig. 1c). Myosin-1B displayed relatively weak affinity for mono-phosphorylated phosphoinositides but demonstrated stronger binding to multiply phosphorylated (Figs. 1d–i). Conversely, myosin-1C showed highest affinity for mono-phosphorylated phosphoinositides, with reduced binding affinity to di- and tri-phosphorylated variants (Figs. 1d–j). Myosin-1D displayed a markedly distinct binding profile, with low affinity for mono-phosphorylated phosphoinositides and a pronounced preference for PI(3,4,5)P₃ (Figs. 1d–j). An almost reciprocal pattern was observed in the affinity constants for individual PIPs between myosin-1C and myosin-1D, correlating with the increasing phosphorylation state of the PIP species (Fig. 1k). Collectively, these data reveal that the affinity of class I myosins for phosphoinositides varies by up to 6- to 20-fold, reflecting both conserved and isoform-specific recognition patterns within their TH1 domains. Such differential phosphoinositide binding reflects a key determinant of specific membrane association, a hypothesis we further investigated through structural modelling.

**Table 1:** Equilibrium dissociation constants of myosin-1 tail binding to phospholipids

Tail construct			
Lipid species	Myosin-1B-tail	Myosin-1C-tail	Myosin-1D-tail
	K_D_, µM		
PI	38.2 ± 4	5.4 ± 0.8	11.5 ± 2.2
PC, PS	> 100	31.5 ± 2.7	33.7 ± 4.5
PI(3)P	8.3 ± 1	4 ± 0.3	1.7 ± 0.3
PI(4)P	2.3 ± 0.4	0.4 ± 0.1	1.2 ± 0.2
PI(5)P	7.7 ± 0.6	0.5 ± 0.1	2.3 ± 0.2
PI(3,4)P_2_	2.2 ± 0.4	0.9 ± 0.2	1.1 ± 0.2
PI(3,5)P_2_	0.9 ± 0.1	0.6 ± 0.1	0.8 ± 0.1
PI(4,5)P_2_	0.9 ± 0.1	1.1 ± 0.2	0.7 ± 0.1
PI(3,4,5)P_3_	1.1 ± 0.2	2.5 ± 0.3	0.3 ± 0.04

### PH domain structure predicts lipid specificity of long-tailed myosin-1s

The phosphoinositide binding profiles of the tails suggest that the myosins discriminate between PIP species, most likely through a stereospecific molecular recognition within the PIP-binding site of their TH domains [17, 22]. Generally, TH domains adopt a fold similar to PH domains, characterized by a conserved core structure comprising an orthogonally arranged seven-stranded β-sandwich [22, 63]. Within this framework, clusters of lysine and arginine residues—particularly spanning the β1, β2, and β3 strands and the β1–β2 loop—coordinate phosphate interactions, with a consensus sequence mediating high-affinity binding to PI(3,4,5)P₃ [25, 64, 65]. However, ligand specificity cannot be reliably predicted based solely on sequence motifs, and the molecular determinants remain incompletely understood [66, 67]. For example, phosphoinositide selectivity has been linked to variations in β1–β2 loop length and amino acid composition of the β3 and β4 strands [64, 68, 69], yet universal rules through which specificity can be deduced, have not been established [65].

**Fig. 2:**
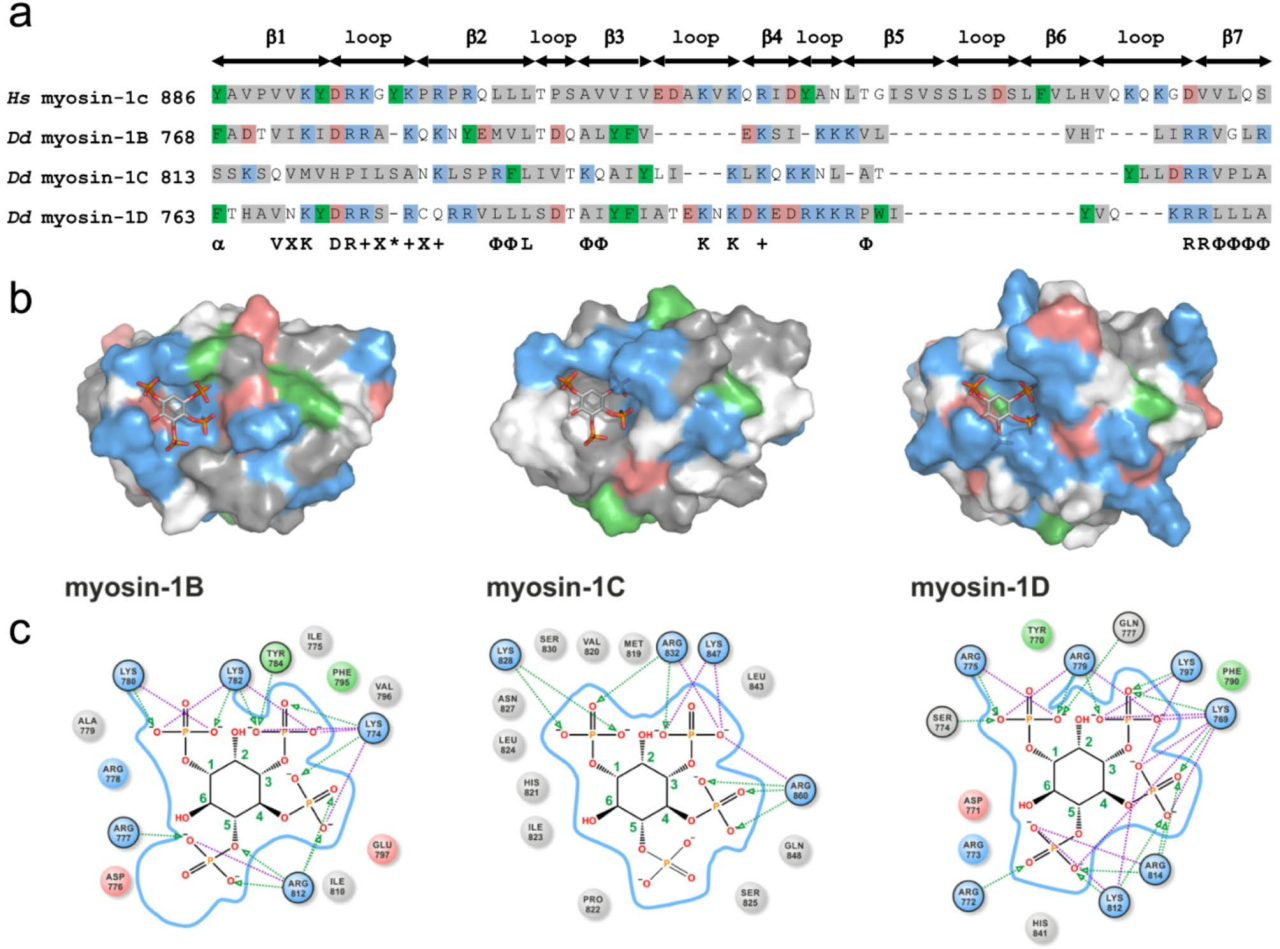
Sequence alignment and homology models of myosin-1 PH domains. **a** Sequence alignment of the PH domains from three *D. discoideum* myosin-1 isoforms with human myosin-1C, color-coded as indicated. The conserved consensus sequence for phosphoinositide (PIP) binding is highlighted in bold below the alignment. **b** Surface representations of the myosin-1B, −1C, and − 1D PH domains modelled in complex with PI(3,4,5)P₃ (depicted as stick models; upper panels). **c** Close-up views of the PI(3,4,5)P₃ binding pockets and coordination spheres. Salt bridges are indicated by purple dotted lines, and hydrogen bonds by green dotted arrows. Protein surfaces and residues are color-coded by amino acid type: positively charged (blue), negatively charged (red), aromatic (green), and nonpolar (gray)

Multiple sequence alignments comparing *Dictyostelium* myosin-1B, −1C, and − 1D with mammalian myosin-1C (Fig. 2a), as well as other myosin-1 isoforms and well-characterized PI(3,4,5)P₃-binding proteins (Supplementary fig. S1), revealed conserved features of the PIP-binding site alongside critical substitutions and variations that likely underlie differential lipid affinities. Notably, myosin-1D—exhibiting the highest affinity for PI(3,4,5)P₃—retains the complement of conserved basic residues essential for coordination of three phosphates within the inositide moiety. In contrast, myosin-1B contains a negatively charged residue within the β2 strand that could electrostatically repel the phosphate, thereby reducing binding affinity. Similarly, myosin-1C lacks a key lysine residue in the β1 strand, potentially weakening its interaction with PI(3,4,5)P₃. Furthermore, variations in the length and amino acid composition of the β1–β2 loop—located proximal to the membrane interface—may contribute to isoform-specific differences in both phosphoinositide specificity and promiscuity.

To corroborate the assumption of an isoform-specific PIP recognition pattern of the TH domain, we employed an in silico approach combining homology modelling and molecular docking to obtain complexes of the individual PH domains with bound PIP3. The crystal structures of PDK1 [70] and the PH domain of human myosin-1C with bound PI(3,4,5)P₃ [61] were used as templates (Fig. 2b). All isoforms exhibited a conserved overall fold predicting a conserved orientation of PIP₃ in the binding pocket; however, the number and nature of residues involved in coordinating the phosphoinositide moiety varied substantially (Fig. 2c). Notably, the myosin-1D PH domain contains seven basic residues, alongside one acidic and one polar residue, implicated in direct PIP3 coordination, whereas myosin-1B and −1C primarily use basic residues for PIP3 complexation. The different PIP interaction profiles correlate well with the experimental data, consistent with myosin-1D displaying the highest PI(3,4,5)P₃-binding affinity and myosin-1C the lowest (Table 1). These findings are further supported by analogous substitutions in mammalian myosin-1b and − 1e, which also exhibit relatively weak PIP₃ binding. Additionally, variations in the length and sequence composition of the β1–β2 loop may further influence both the specificity and promiscuity of PIP binding across isoforms.

Together, these data support a model in which subtle sequence variations within the phosphoinositide-coordinating site govern PIP binding affinity, implying that the PH domain serves as a major determinant for isoform-specific membrane association. Thus, expression levels of the proteins and the relative amount of single PIP species within a membrane are expected to influence the localization profile.

### Competitive phosphoinositide interactions reveal fractional distribution of myosin-1s with membranes

**Fig. 3:**
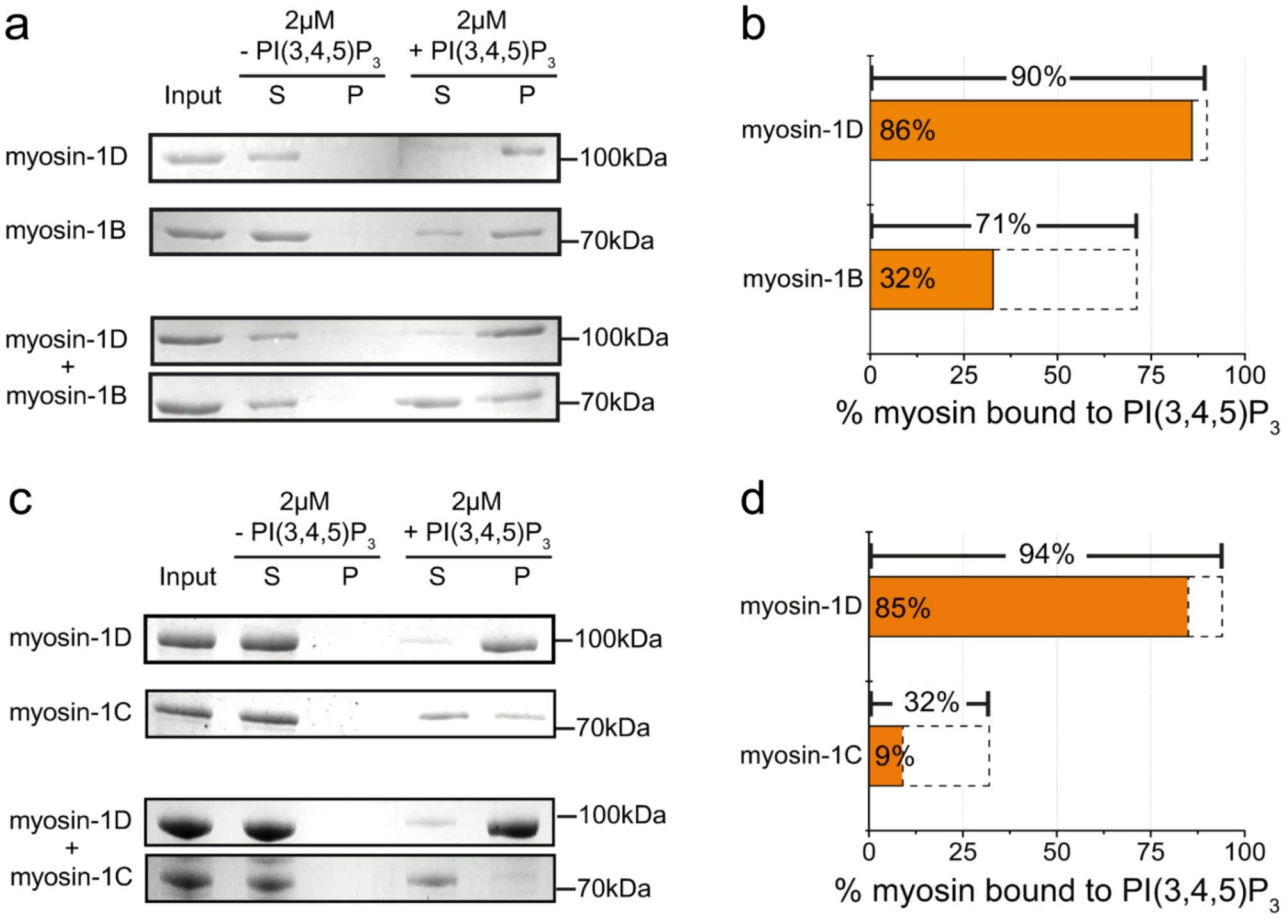
Competitive binding of myosin-1 tails to PI(3,4,5)P₃. **a**, **c** Western blots of supernatant and pellet fractions from lipid vesicles containing PI(3,4,5)P₃ following incubation with 2 µM myosin-1B tail (~ 70 kDa) (**a**) or myosin-1C tail (~ 70 kDa) (**c**) in competition with myosin-1D tail (~ 100 kDa). The total accessible PI(3,4,5)P₃ concentration was maintained at 2 µM. **b**,** d** Dashed boxes indicate the fraction of myosin-1 tails bound to lipid vesicles in the absence of the other myosin isoform. Orange bars denote the fraction of bound myosin in the presence of the competing myosin

To gain deeper insight into the binding specificity of the myosin tails, we conducted competition experiments in which two tail constructs were simultaneously present in the PIP-binding assay, allowing us to assess their ability to compete for binding to the same lipid species. We performed these experiments using PI(3,4,5)P₃ (PIP₃) as an exemplary lipid, first in the absence and then in the presence of a second tail isoform. Two tail pairs were tested: those of myosin-1B and myosin-1D, and those of myosin-1C and myosin-1D (Figs. 3a, c). At equimolar concentrations (2 µM), incubation of a single tail with PIP₃-containing liposomes resulted in approximately 90–94% bound myosin-1D, 71% bound myosin-1B, and 32% bound myosin-1C (Figs. 3b, d). In contrast, when both isoforms were present, the fraction of myosin-1D bound remained largely unchanged (85% and 86%, respectively), whereas binding of myosin-1B and myosin-1C was reduced to 32% and 9%, respectively; consistent with their relative lipid affinities (Table 1). These findings establish myosin-1D as the dominant binder of PIP₃, capable of outcompeting myosin-1B and myosin-1C for membrane association.

**Fig. 4:**
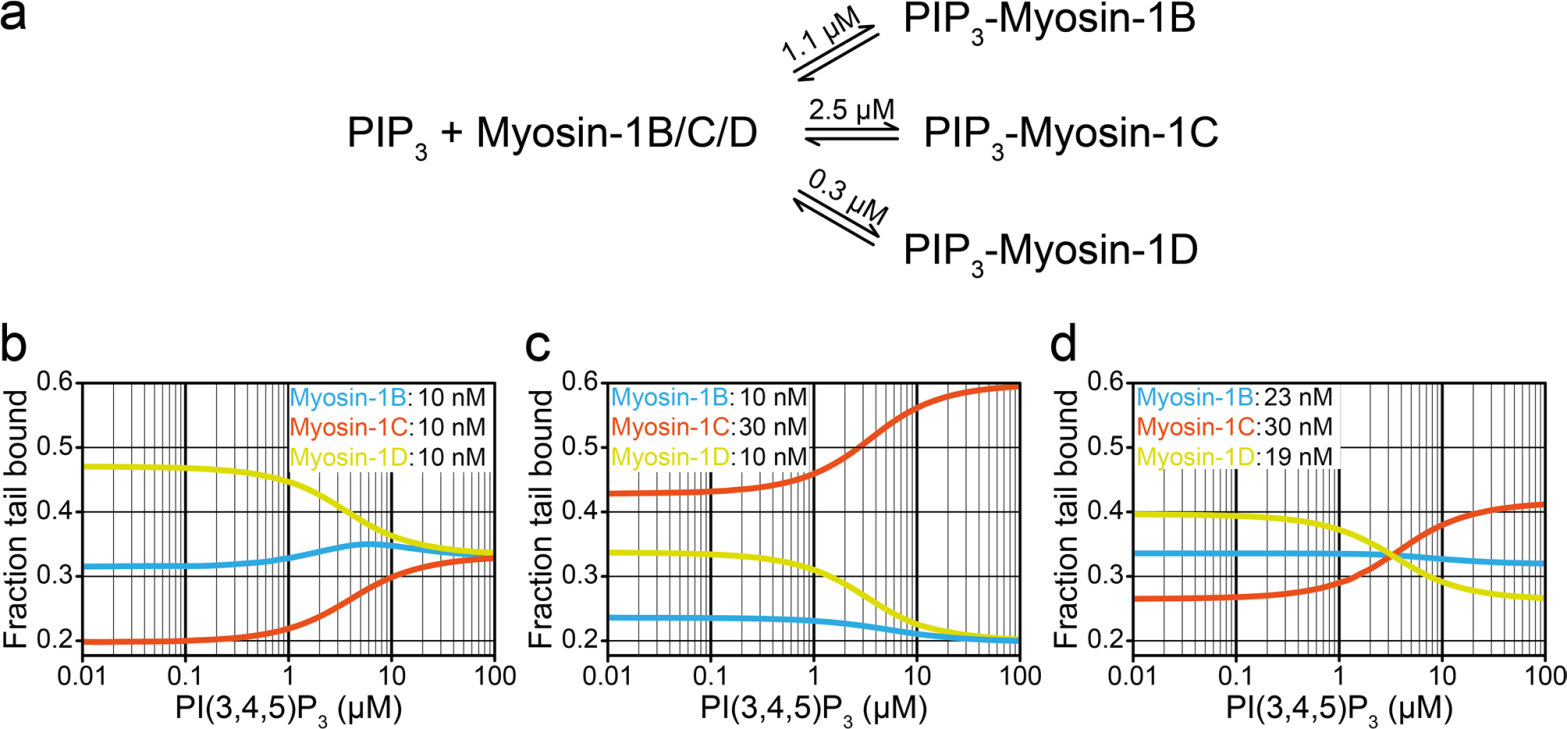
Kinetic modelling of PIP3-myosin-1 binding equilibria. **a** parallel linear equilibrium reaction scheme of the PIP_3_-Myosin-1 complex formation. The graphs represent three scenarios: **b** myosins are in equimolar presence, **c** the low-affinity binder is in a threefold excess relative to the others, and **d** concentrations vary slightly.

This competitive binding suggests that the association of myosin isoforms with membranes may dependent on the relative abundance of the PIPs within the membrane. We simulated such a competitive binding exemplary for PIP₃ using a parallel linear equilibrium model (Fig. 4). Incorporating the experimentally determined affinity constants to a physiologically relevant range of PIP concentrations [71–73], we modeled three scenarios: [1] equimolar presence of the myosins (Fig. 4b) [2], a threefold excess of the low-affinity binder relative to the others (Fig. 4c), and [3] slightly varying myosin concentration to mimic differential expression levels observed in vivo (Fig. 4d). Under equimolar conditions, myosin-1D dominated PIP₃ binding, while myosin-1B and myosin-1C showed up to a twofold reduction in PIP₃ occupancy. However, this dominance decreased at higher PIP₃ concentrations. Since intracellular PIP₃ levels can increase substantially upon stimulation, reaching micromolar concentrations [74], binding to a single PIP species may become more evenly distributed among myosins, with each isoform occupying roughly one third of the available sites. Notably, when myosin-1C is present at a threefold excess, it outcompetes the other isoforms across the entire concentration range (Fig. 4c). Furthermore, minor variations in isoform expression levels, as previously documented for these myosins [52], can invert their relative PIP₃ binding affinities (Fig. 4d).

These findings highlight that both, fluctuations in phosphoinositide levels and myosin isoform abundance may critically modulate membrane association dynamics of the motors. As a result, myosins can either share the same membrane localization or a single isoform will dominate, depending on intracellular regulatory cues that influence PIP concentrations and myosin expression. Fine-tuning the relative abundance of myosin-1 isoforms in relation to PIP levels thus represents a versatile mechanism for more precise regulation of membrane targeting.

### Phosphoinositides mediate distinct myosin-1 membrane associations during endocytosis

**Fig. 5:**
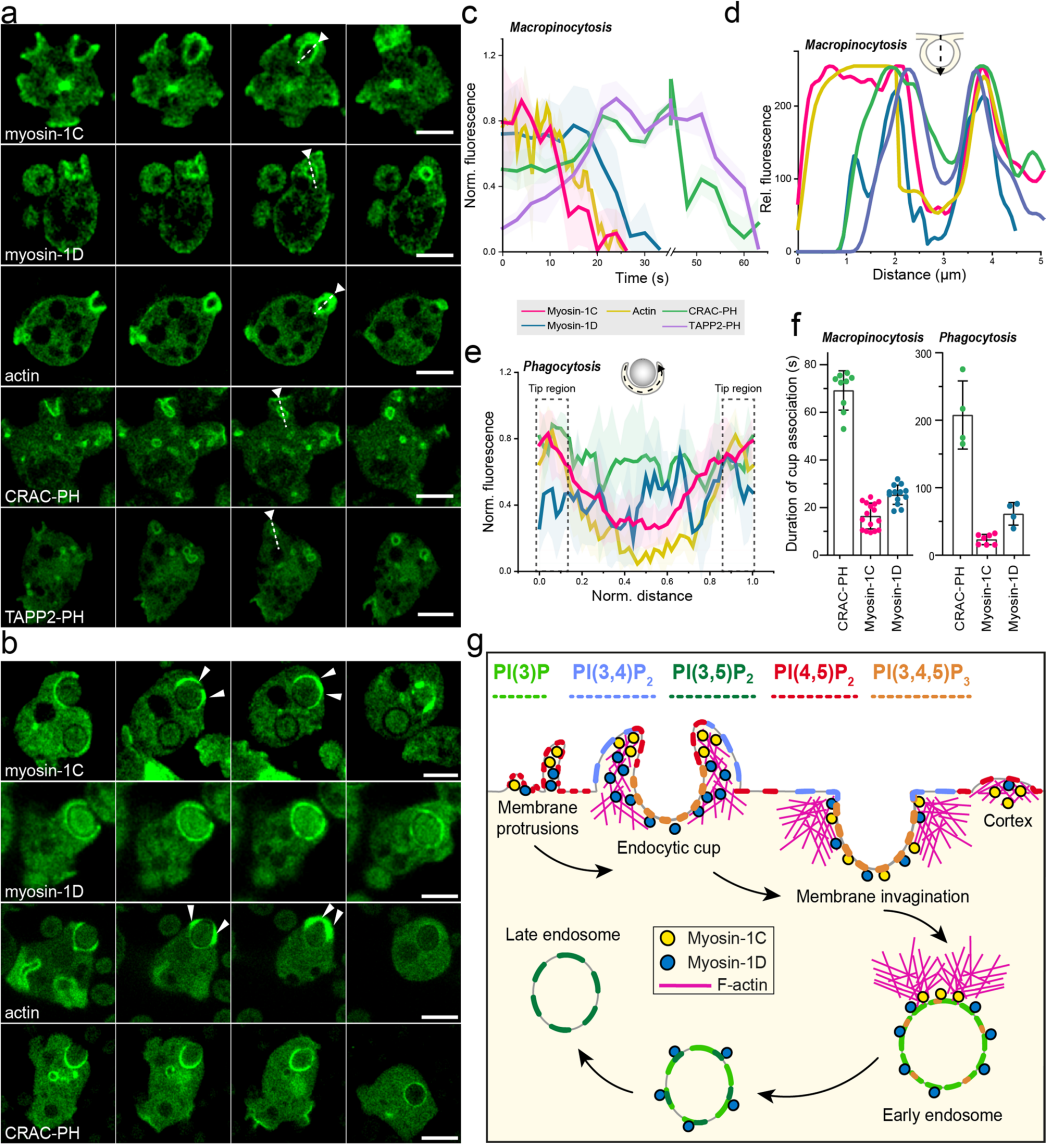
Myosin-1C, myosin-1D, CRAC-PH, and TAPP2-PH dynamics during macropinocytosis and phagocytosis of Dictyostelium cells. **a** Confocal time-lapse images showing the localization of Myosin-1C, Myosin-1D, F-actin, CRAC-PH, and TAPP2-PH during macropinocytosis. **b** Confocal time-lapse images showing the localization of the same proteins during phagocytosis. **c** Temporal fluorescence intensity profiles of Myosin-1C, Myosin-1D, F-actin, CRAC-PH, and TAPP2-PH measured along cross-sections spanning the plasma membrane and endosome following macropinosome closure. Scale bars, 5 μm. **d**,** e** Fluorescence intensity profiles of endosomal scission events during (**d**) macropinocytosis and (**e**) phagocytosis, as illustrated schematically. **f** Quantification of the duration of cup association of myosin-1C and myosin-1D during macropinocytic and phagocytic events. Data were tested for normality with a Shapiro-Wilk test and then p values were determined with a Mann-Whitney test (Macropinocytosis) or unpaired t-test (Phagocytosis). Data represent averages ± S.D. **g** Schematic model illustrating phosphoinositide-dependent recruitment of myosin-1 isoforms along the endocytic pathway. During endocytosis, myosin-1C and myosin-1D are recruited to membrane protrusions enriched in PI(3,4)P₂ and PI(4,5)P₂ at endocytic cups containing PI(3,4,5)P₃ produced by PI3-kinase activity. As endocytosis proceeds, myosin-1D remains associated with the endosome, whereas Myosin-1C localizes also to the cortical membrane. Post-endosome formation, PI(3)P becomes the dominant phosphoinositide species, with myosin-1D being associated. Late, P(3,5)P_2_ enriched endosomes are devoid of myosin-1s

To extend our biochemical findings into the cellular context, we employed confocal fluorescence microscopy and examined the spatiotemporal dynamics of the myosins during large-scale fluid-phase endocytosis (macropinocytosis) and phagocytosis—processes reliant on cortical actin remodeling and plasma membrane invagination to form nutrient- and particle-containing endosomal vesicles (Fig. 5). Myosin-1 isoforms have been extensively characterized in *Dictyostelium*, with myosin-1B being the most studied localizing uniformly along the plasma membrane of resting cells, accumulating at active protrusions and cell–cell contacts during cell migration, and associating with actin at the leading edge of polarized cells [75] along ventral actin waves and the plasma membrane, where it is implicated in anchoring actin waves via interactions with PIP2/PIP3 through the TH1 domain, and with F-actin via the Gly–Pro–Gln-rich tail region [75–77] primarily involved in tension during migration. In contrast myosin-1C and myosin-1D have been more prominently associated with endocytic processes [23]. Our confocal imaging of fixed AX2 cells expressing YFP-variants of the proteins, including the short-tailed myosin-1E, confirmed these reported localization patterns of the myosins: myosin-1B predominantly localized at protruding fronts with actin waves, whereas myosin-1E, myosin-1C, and myosin-1D associated very pronounced with macropinocytic structures (Supplementary fig. S4a).

Since the localization profiles of myosin-1B and myosin-1E have been well documented, we focused our analysis on the less-studied myosin-1C and myosin-1D. In *Dictyostelium* cells undergoing starvation, a condition known to increase expression levels of the proteins that promotes pino- and phagocytosis [15, 50, 78, 79], myosin-1C and myosin-1D localized to macropinocytic structures. Both myosins were enriched along the membrane of the sealing pinosome (Fig. 5a), showing patterns similar to their short-tailed homologues myosin-1E and myosin-1 F [80]. Myosin-1C exhibited localization dynamics that closely paralleled cortical actin, being prominent at the cell cortex and during phagocytic cup formation but largely absent from maturing endosomes. Myosin-1D demonstrated a prolonged association with endosomes and remained present on internalized macropinocytic vesicles after cup closure. To correlate myosin localization with phosphoinositide dynamics, we used the fluorescent reporters CRAC-PH and TAPP2-PH to monitor PI(3,4)P₂/PI(3,4,5)P₃ and PI(3,4)P₂ distributions, respectively [81]. During macropinocytosis, CRAC-PH remained enriched at the plasma membrane and surrounded the closing cup, whereas TAPP2-PH localized more prominently around the internalized pinosome and less at the plasma membrane (Fig. 5a), consistent with previous reports [81].

Given the mechanistic similarities between macropinocytosis and phagocytosis, both characterized by conserved clathrin-independent phosphoinositide dynamics [82], we examined myosin-1 localization during yeast uptake. Unlike macropinocytic cups, which form circular, ruffled protrusions generating large liquid-filled vesicles, phagocytic cups are typically asymmetric and conform to the shape of the particle. Myosin-1C localized along the rim of the phagocytic cup, accumulating at the distal tips during particle engulfment, and disappeared from the internalized vesicle shortly after cup closure (Fig. 5b; Movies M2). This dynamic closely resembles that observed during macropinocytosis and paralleled actin dynamics almost completely.

Fluorescence intensity analyses corroborated this similarity, revealing that myosin-1C associates transiently with the macropinosomal membrane mirroring actin dynamics. In contrast, myosin-1D exhibits a more sustained localization resembling that of the PI(3,4,5)P₃-binding probes CRAC-PH and TAPP2-PH (Figs. 5c, d). Myosin-1D remained associated with the cup throughout the entire phagocytic process and surrounded almost completely the entire membrane of the internalized phagosomes. This association persisted for a longer duration compared to myosin-1C, which showed a shorter localization at the cup before becoming enriched at the cortex (Fig. 5e; Movies M1,M2). Quantitative analysis confirmed that myosin-1D remained associated with macropinocytic and phagocytic endosomes by up to 1.5- and 2.5-fold longer than myosin-1C, respectively (Fig. 5d). Interestingly, myosin-1D dissociated from the maturing pinosome earlier than CRAC-PH and TAPP2-PH (Figs. 5c, d, f), suggesting that additional mechanisms contribute to the regulation of its release during vesicle maturation that are independent of PI(3,4)P₂ and PI(3,4,5)P₃ dynamics.

These localization patterns suggest that myosin-1C and myosin-1D may have different, stage-specific roles during endocytosis, influenced by phosphoinositide composition. Both associate with PI(4,5)P₂-rich, actin-positive membrane ruffles during early phagocytosis of cup formation, with PI(3,4,5)P₃ prevailing on early endosomes. Myosin-1D’s higher affinity for PI(3,4,5)P₃ supports its persistence on the internalized vesicles as phosphoinositide composition shifts from PI(3,4,5)P₃ to PI(3)P, for which it also displays a greater binding preference. By contrast, myosin-1C’s stronger affinity for actin may disrupt the endosomal association, favouring interaction with actin-rich structures, which support distinct cellular functions during vesicle maturation.

### PI(3,4,5)P₃ depletion disrupts endosomal localization of long-tailed myosin-1s

The observed similarity in localization between the myosins with the PIP reporters CRAC-PH and TAPP2-PH, respectively, most prominently for myosin-1D, suggests a predominant role of PI(3,4)P₂ and PI(3,4,5)P₃ in mediating the recruitment of these myosins to cortical and endosomal membranes, albeit with slightly different lipid preferences. Building on the working hypothesis that the conversion of PI(4,5)P₂ to PI(3,4,5)P₃ during the dynamic processes of phagocytic cup extension and subsequent endosome formation serves to stabilize the association of myosin-1D and myosin-1C with the endosomal membrane, we predicted that experimental depletion of PI(3,4,5)P₃ would disrupt localization. This hypothesis is based on the idea that the generation of PI(3,4,5)P₃ creates a lipid microenvironment that anchors myosins at the sites of active membrane remodelling, thereby facilitating membrane deformation and trafficking events required for phagosome formation.

**Fig. 6:**
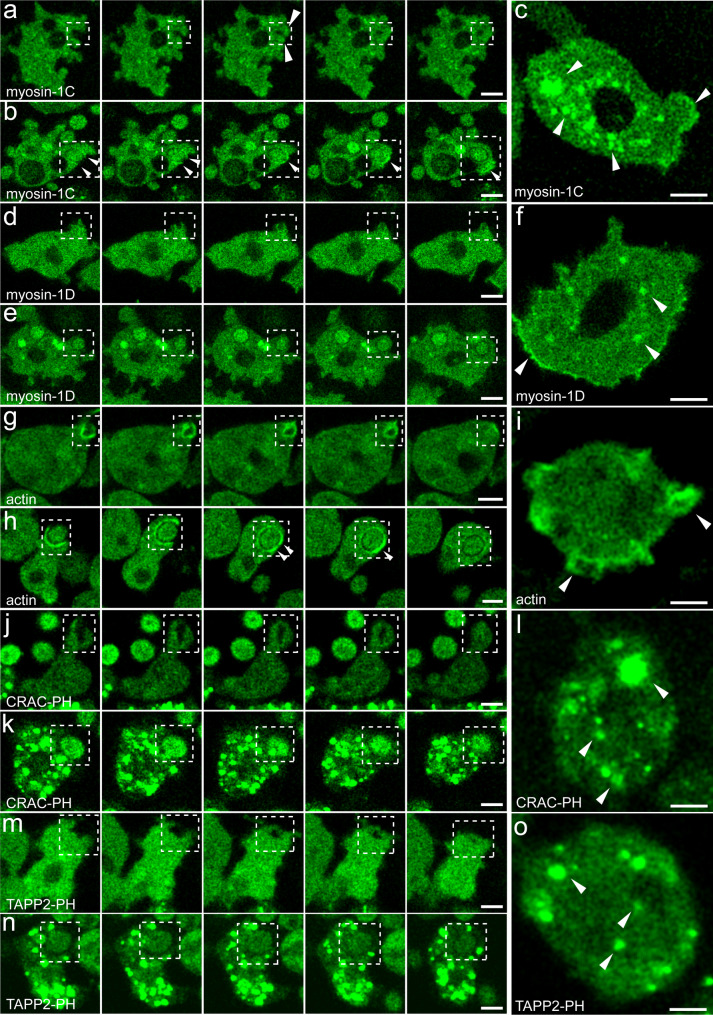
Myosin-1 localization in PI(3,4,5)P₃-depleted cells during endocytosis. Confocal time-lapse series of *D. discoideum* cells treated with the PI3K inhibitor LY294002, showing the subcellular localization of myosin-1C (**a-c**), myosin-1D (**d-f**), F-actin (**g-i**), CRAC-PH (**j-l**), and TAPP2-PH (**m-o**) during fluid-phase macropinocytosis and yeast phagocytosis. Arrows indicate sites of strongest membrane association. Scale bars, 5 μm

To experimentally test this, *Dictyostelium* cells undergoing starvation were treated with 20 µM LY294002 [83], a PI3-kinase inhibitor known to effectively reduce membrane levels of PI(3,4,5)P₃ without broadly impairing the overall capacity of cells to carry out endocytic uptake, making it a valuable tool for dissecting PI(3,4,5)P₃-dependent processes [84, 85]. Following treatment, although cells retained the ability to form protrusions, invaginations, and release endosomes (Fig. 6), the localization of myosin-1C and myosin-1D with endocytic cups and endosomal membranes was almost fully abolished (Movies M3, M4**;** Figs. 6a, b, d, e). Instead of the typical membrane association, both isoforms exhibited diffuse cytoplasmic distribution, except for myosin-1C, which retained a partial membrane association. Moreover, the myosins accumulated in discrete vesicle-like structures that were positive for CRAC-PH and TAPP2-PH (Figs. 6 c, f, j–o), suggesting a shift in their membrane-binding preferences or availability of PIP-species. Critically, LY294002 treatment did not disrupt the typical cortical actin dynamics or the close association of actin with the early endosome (Figs. 6g–i), indicating that the mislocalization of the myosins is largely independent of actin and dominated by the increased PI(3,4,5)P₃ levels.

This highlights the crucial role of PI(3,4,5)P₃ in coordinating the spatial positioning of myosin-1 isoforms at membranes undergoing remodelling. The residual membrane targeting implies that myosin-1 isoforms can also interact with other phosphoinositide species, such as mono-phosphorylated or dephosphorylated inositides, consistent with the above documented affinities for the distinct lipid species. Such versatility in lipid recognition may allow myosins to maintain a basal level of membrane association under varying phosphoinositide conditions, thereby supporting their functions in membrane trafficking and cytoskeletal dynamics.

### Tail-mediated actin binding facilitates myosin-1 association with cortical membranes

**Fig. 7:**
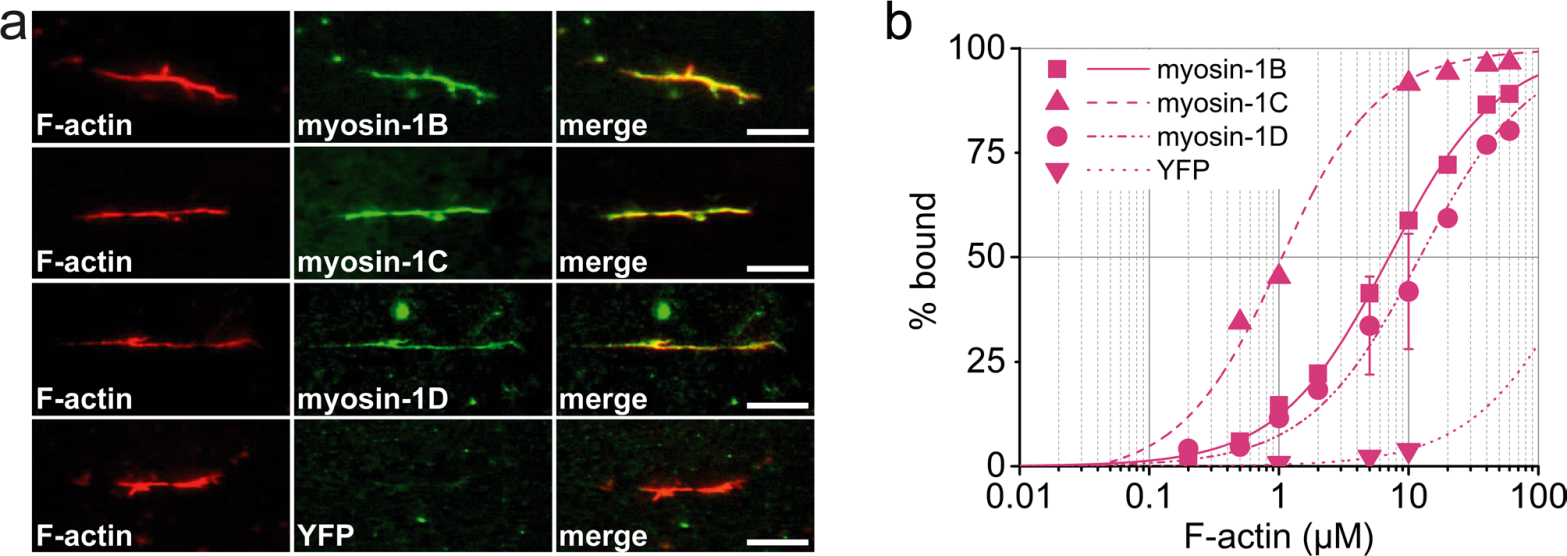
Myosin-1 tail binding to F-actin. **a** Representative fluorescence micrographs depicting direct binding of purified myosin-1 tails (green) to surface-immobilized F-actin filaments (phalloidin-labeled, red). Scale bar, 10 μm. **b** Half-logarithmic binding curves illustrating the fraction of myosin-1 tail bound to F-actin over a range of increasing filament concentrations. Purified YFP served as a negative control to assess nonspecific binding

Beyond tail-mediated phosphoinositide binding, the interaction of myosin-1 isoforms with F-actin via their GPQ-rich tail regions has been proposed as a complementary mechanism facilitating cortical membrane association. This actin-binding is thought to work alongside the motor domain’s interaction with actin filaments to stabilize membrane localization [86, 87]. However, the actin-binding properties of myosin-1 tails remain incompletely characterized, with most previous studies focusing primarily on other isoforms [75–77, 80, 88–92]. To elucidate the contribution of an ATP-insensitive actin-binding mechanism to myosin-1 localization, as previously reported for myosin-1B [75], we employed total internal reflection fluorescence (TIRF) microscopy. Using YFP-tagged tail constructs, we directly visualized myosin-1 tail binding to surface-immobilized, TRITC-phalloidin-labeled actin filaments (Fig. 7a). All three YFP-myosin-1 tail isoforms decorated the entire length of actin filaments, whereas a YFP control showed no binding, confirming specificity (Fig. 7a). Notably, quantitative analysis revealed significant differences in binding affinities among isoforms (Fig. 7b). The tails of myosin-1B and myosin-1D exhibited considerably weaker actin binding, with K_D_ values of 6.8 ± 0.2 µM and 12 ± 0.8 µM, respectively—approximately seven- and twelve-fold weaker compared to the myosin-1C tail, which showed a high-affinity interaction (K_D_ = 0.9 ± 0.4 µM) (Table 2).

**Table 2:** Equilibrium dissociation constants of myosin-1 tail binding to F-actin and microtubules and inhibitory properties of myosin-1-tails on microtubule depolymerization

	Tail construct				
	Myosin-1B	Myosin-1C	Myosin-1D	Myosin-1D-ΔSH3	Myosin-1D-TH1
	K_D_, µM				
F-actin	6.8 ± 0.2	0.8 ± 0.08	12 ± 0.8	n.d.	n.d.
Microtubules	0.9 ± 0.1	0.08 ± 0.05*	0.3 ± 0.1	0.4 ± 0.1	3.3 ± 0.2
Inhibition of the cold-induced depolymerization of microtubules IC _50_, nM					
	n.a.	2.4 ± 1 nM	15 ± 4 nM	n.d.	n.d.

* Rump et al., 2010; n.a.: not applicable; n.d. not determined

These differences in actin affinity may underlie the distinct localization dynamics observed during endosome formation. Myosin-1C’s strong affinity for F-actin likely facilitates its retention at the actin-rich cortex and its gradual disappearance from one side of the endosomal membrane as the endosome matures. Early endosomes are typically surrounded by a dense actin network, which supports vesicle motility, positioning, and maturation. Myosin-1C’s ability to tightly bind actin enables it to maintain in close association with the endosome during these early stages. As actin disassembles during maturation, myosin-1C dissociates accordingly.

In contrast, myosin-1D, with its weaker actin affinity, shows prolonged association with the endosomal membrane despite the progressive loss of actin around maturing vesicles. This suggests that myosin-1D localization is predominantly governed by phosphoinositide binding rather than actin interactions. This interpretation aligns with the well-characterized transition from an actin-rich early endosome environment to a late endosome stage, characterized by reduced actin presence and changes in phosphoinositide composition. Myosin-1D’s sustained membrane association during this phase points to a role in vesicle maturation processes that rely less on actin scaffolding and more on lipid signalling cues, emphasizing the importance of phosphoinositide-driven recruitment mechanisms for its function.

### Differential PIP binding and cytoskeletal crosstalk guide myosin-1 membrane shuttling

**Fig. 8:**
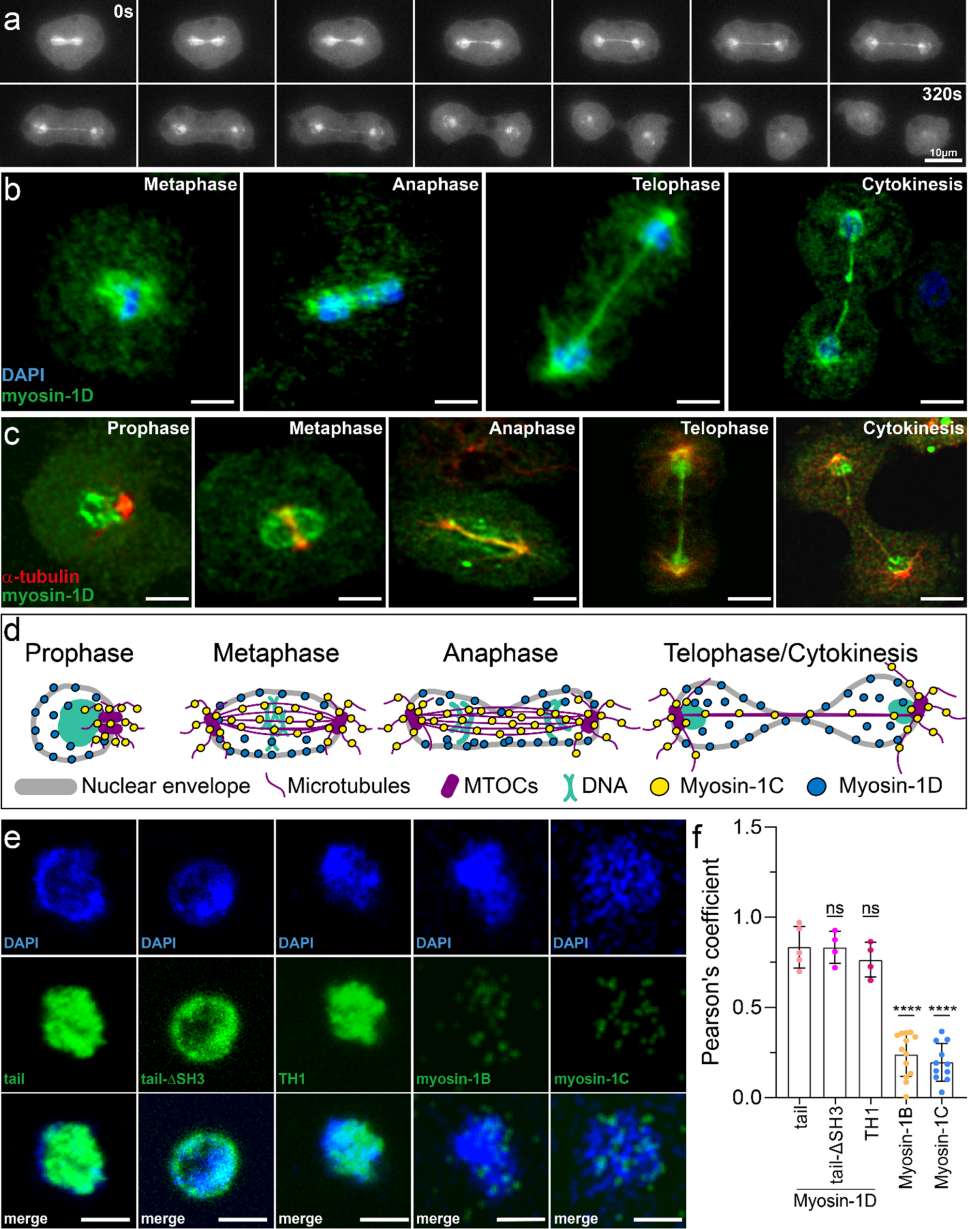
Myosin-1D localization during mitosis. **a** Confocal time-lapse series of YFP-myosin-1D localization in a *D. discoideum* cell during mitosis and cytokinesis. Images acquired every 25 s. **b** Representative confocal images of *D. discoideum* cells during mitosis showing chromatin (blue) and myosin-1D (green). **c** Confocal images depicting myosin-1D (green) and α-tubulin (red) across different mitotic stages. Scale bars, 10 μm. **d** During prophase, myosin-1C associates with microtubules near the microtubule-organizing center (MTOC), while myosin-1D decorates the nuclear envelope. In metaphase and anaphase, myosin-1C localizes to midzone microtubules inside the nuclear envelope and along astral microtubules outside it. Myosin-1D remains associated with the nuclear membrane and partially with microtubules. In telophase, as spindle pole folding continues and the nuclear envelope fenestrates, exposing spindle microtubules, myosin-1D remains on the nuclear envelope and colocalizes with spindle microtubules. Concurrently, myosin-1C stays tightly bound to spindle and astral microtubules. During cytokinesis, as the cleavage furrow forms and spindle microtubules disassemble, both myosin-1C and myosin-1D gradually disappear from the spindle and nuclear membrane, respectively. **e** Confocal z-slice projections showing binding of YFP-tagged myosin tail constructs to isolated nuclei stained for chromatin (blue). Full-tail myosin-1B and myosin-1C constructs do not bind to nuclei. Scale bars, 2 μm. **f** Pearson’s coefficient-based analysis of fluorescent signals reveals for myosin-1D a correlation, confirming specific binding. Data were tested for normality with a Shapiro-Wilk test and then p values were determined with an unpaired t-test. Data represent averages ± S.D

We previously demonstrated that myosin-1C contributes to spindle integrity and faithful chromosome segregation during mitosis by relocating from the plasma membrane to the mitotic spindle, where it remains associated throughout division to support spindle stability and anchorage [50]. Interestingly, myosin-1D exhibited a similar cell cycle–dependent redistribution during transition from interphase into mitosis and subsequent mitotic events (Fig. 8a). However, in contrast to myosin-1C, we observed myosin-1D to localize at the nucleus, indicative of an association with the nuclear envelope and/or condensed chromatin masses. This association was maintained throughout mitosis including cytokinesis (Fig. 8b, Movie M5). At telophase and during cytokinesis, apart from decorating the entire chromatin masses, myosin-1D also displayed a partial colocalization with central microtubules and microtubules emerging from the spindle poles (Fig. 8c, Supplementary fig. S6a, c,d). The localization of myosin-1D differed notably from that of myosin-1C, being predominantly concentrated around or with chromatin masses [50]. To further investigate the spatial association of myosin-1D with the nucleus, we employed the kinesin-related protein K7, which, besides its microtubule-binding function, has been reported to localize to a membranous perinuclear structure, likely corresponding to the nuclear envelope complex [93]. In our experiments, K7 signal was detected in perinuclear regions (Supplementary fig. S6b), consistent with localization to the ER–nuclear envelope membrane system. Orthogonal XZ and YZ projections confirmed this perinuclear localization, as previously described; however, myosin-1D showed only partial overlap with K7, occupying a volume within the nuclear boundaries. While a perinuclear membrane association might be a feature of myosin-1D, the predominant intranuclear localization potentially reflects the principal functional role of myosin-1D. The schematic in Fig. 8d summarizes the localization patterns of myosin-1C and myosin-1D observed during mitosis, highlighting myosin-1C’s exclusive association with components of the mitotic spindle and myosin-1D’s enrichment.

The prominent nuclear localization of myosin-1D during cell division prompted us to investigate the molecular basis for this association. *Dictyostelium* undergoes a closed mitosis, during which the nuclear envelope remains intact throughout the mitotic process [94], suggesting that myosin-1D may target the nuclear membrane via lipid interactions mediated by its TH1 domain. To test this, we generated a series of YFP-tagged myosin-1D tail constructs, including the full-length tail, a variant lacking the SH3 domain (tail-ΔSH3), and a truncated version lacking both the TH2 and SH3 domains. The SH3 domain truncation constructs were selected to investigate potential loss of nuclear binding, given that the SH3 domain in myosin-1D—unlike in myosin-1C—is positioned between the TH1 and TH2 domains, which may affect lipid binding properties. Additionally, the SH3 domain is known to mediate protein-protein interactions, potentially contributing to nuclear localization, a hypothesis we sought to examine. Using isolated, membrane-enclosed nuclei from vegetatively grown AX2 cells, we evaluated the nuclear binding capacity of all constructs (Fig. 8e). The results revealed robust decoration around the nuclear surface, indicating that the TH1 domain alone is sufficient to mediate nuclear envelope association, consistent with its phosphoinositide-binding capability. In contrast, YFP-tagged myosin-1B and myosin-1C constructs containing the full tail did not associate with the nuclei, as confirmed by colocalization analysis using Pearson coefficient determination (Fig. 8e). While these findings do not rule out additional nuclear interactions facilitated by e.g. SH3-dependent binding to nuclear partners, they support a predominant role for TH1 domain–mediated binding to the nuclear membrane.

**Fig. 9:**
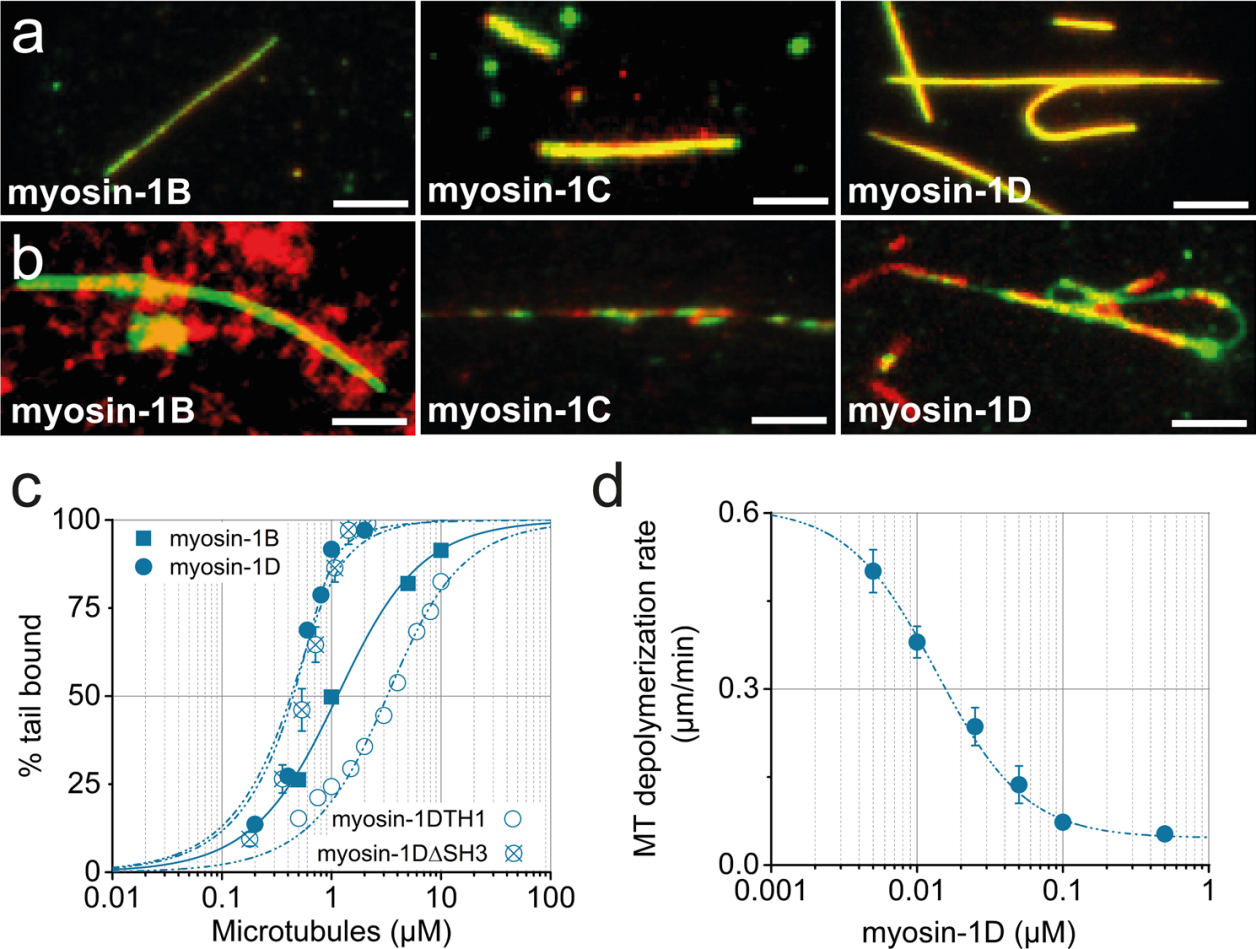
Myosin-1 binding to microtubules. **a** TIRF microscopy images demonstrating direct binding of myosin-1 tails (red) to surface-immobilized microtubules (green). Scale bars, 10 μm. **b** TIRF microscopy images showing microtubules (green) and F-actin (red) following incubation with 1 µM myosin-1 tail constructs. Merged images reveal crosslinking activity of myosin-1D and myosin-1C. **c** Half-logarithmic plots illustrating binding affinities of myosin-1 tail constructs to microtubules. Equilibrium dissociation constants were derived from hyperbolic curve fitting. **d** myosin-1D tail–mediated inhibition of cold-induced microtubule depolymerization. Kinetics exhibit hyperbolic dependence with a half-maximal inhibitory concentration (IC_50_) of 15 ± 4 nM. Error bars represent SD from three independent experiments

Since myosin-1D colocalized also with microtubules starting from anaphase (Fig. 8c), we next investigated the ability of the myosin-1D tail to directly interact with microtubules, as previously shown for myosin-1C [50]. Binding and sedimentation assays revealed that the myosin-1D tail binds microtubules with high affinity (K_D_ = 0.3 ± 0.09 µM), comparable to myosin-1C (Figs. 9a, c). This interaction persisted in the SH3 domain deletion construct (myosin-1D-tail-ΔSH3; K_D_ = 0.4 ± 0.01 µM), whereas the isolated TH1 domain exhibited significantly weaker binding (K_D_ = 3.3 ± 0.1 µM; Table 2). Beyond binding, the myosin-1D tail stabilized microtubules in a concentration-dependent manner, with an IC₅₀ of ~ 20 nM in cold-induced depolymerization assays (Fig. 9d), compared to ~ 2 nM reported for myosin-1C [50]. Single molecule tracking of myosin-1C and myosin-1D tails on microtubules revealed diffusional movement, as shown by kymograph analysis (Supplementary fig. S5), consistent with transient binding. Furthermore, co-incubation of microtubules and actin filaments with either myosin-1C or myosin-1D tails prior to immobilization revealed cross-linking activity, a property not observed for myosin-1B (Fig. 9b).

## Discussion

Our study reveals critical insights into the coordination of membrane–cytoskeleton interactions by class-1 myosins, mediated through their tail domains via both shared and isoform-specific phosphoinositide recognition. We demonstrate that subtle sequence variations within the conserved PH-like tail homology (TH) domains impart distinct lipid-binding specificities among the long-tailed isoforms, enabling differential membrane targeting. Specifically, myosin-1D exhibits a high affinity for PI(3,4,5)P₃ and PI(3)P, myosin-1C preferentially binds mono-phosphorylated phosphoinositides, with strong affinity for PI(4)P and PI(5)P but weaker binding to PI(3)P. In contrast, myosin-1B displays relatively weak affinity for mono-phosphorylated PIPs compared to the other isoforms but shows comparable affinity for PI(3,5)P_2_ and PI(4,5)P_2_, the predominant PIP species at the cell cortex where all three myosins localize. These biochemical adaptations underpins an isoform-specific recruitment to dynamic membrane compartments. Structural modelling supports that differences in basic residues and loop conformations within the TH domain drive these binding specificities, accounting for myosin-1D’s robust interaction with PI(3,4,5)P₃ relative to the weaker affinities of myosin-1B and myosin-1C, a finding consistent with emerging paradigms of phosphoinositide recognition by modular lipid-binding domains [95, 96].

Functionally, the distinct lipid-binding preferences of class-1 myosins translate into isoform-specific spatiotemporal behaviors during macropinocytosis and phagocytosis. Consistent with its high actin binding affinity mediated by the tail (Table 2), myosin-1C is enriched at actin-dense plasma membrane regions. In line with its lipid specificity (Table 1), myosin-1C accumulates at the distal tips of PI(3,4)P_2_/PI(4,5)P₂ enriched phagocytic cups. It rapidly dissociates following vesicle internalization (Fig. 5e), supporting a role in early cup formation and membrane–cytoskeleton tethering. This localization pattern correlates with its strong affinity for mono-phosphorylated PIPs and actin [77]. Myosin-1C, myosin-1D, and myosin-1B all display relatively high affinities for membrane protrusions enriched in PI(3,4)P₂, PI(4,5)P₂, and PI(3,4,5)P₃. However, myosin-1D’s sustained association with sealing phagosomes enriched in PI(3,4,5)P₃ and PI(3)P (Fig. 5e) aligns with its higher affinity for these lipids (Table 1), implicating it in later stages of vesicle maturation. This suggests a coordinated response to the dynamic phosphoinositide transitions that orchestrate endocytic progression [97, 98]. Pharmacological inhibition of PI3-kinase propose a recruitment mechanism in which PI(3,4,5)P₃ is essential for the initial membrane association of myosin-1C and myosin-1D at the forming phagocytic cup. For myosin-1C, residual localization to the phagosome persists, likely mediated by its high affinity to cortical actin, elevated expression levels, and/or the relative lipid composition of the membrane. In contrast, myosin-1D is redirected to late endosomal and vesicular compartments in the absence of PI(3,4,5)P₃, suggesting that its localization is less dependent on actin and instead may be driven by PI(3)P and PI(4,5)P₂ enrichment on these structures.

Together, these findings extend recent models of lipid-driven motor localization and highlight the critical role of myosin tail architecture in isoform-specific targeting [99]. The localization patterns defined by lipid specificity and cytoskeletal interactions align with previous studies implicating myosin-1 isoforms as key mediators of membrane tension and cortical actin organization [4]. Our data, interpreted in the context of equilibrium interactions between myosin-1 isoforms and phosphoinositides, further highlight that motor expression levels, lipid-binding affinities, and local membrane composition collectively govern competitive and distinct interactions with membranes and the cytoskeleton among the isoforms. This interdependence may serve as a stage-specific recruitment mechanisms, allowing cells to finely tune motor localization in response to changes in phosphoinositide landscapes. Moreover, membrane association is not solely dictated by phosphoinositide binding but arises from synergistic interactions with F-actin networks. While PIP_x_ binding drives initial motor recruitment, both motor-domain and tail-mediated actin-binding can critically influence retention and duration of cortical distribution. The notably higher actin affinity likely accounts for the sustained cortical presence of myosin-1C after endosome internalization and with actin waves for myosin-1B, distinguishing them from myosin-1D. Furthermore, structural differences in tail domain architecture—such as the atypical internal positioning of the TH3 domain in myosin-1D compared to its canonical C-terminal location in other isoforms may modulate the interplay between lipid binding and protein partners, contributing to the unique nuclear membrane association of this motor. Additionally, single amino acid variations and post-translational modifications should be regarded as relevant mechanisms of regulation of lipid and cytoskeletal interactions as exemplified [100, 101]. Collectively, this refined mechanism advances our understanding of how myosin-1 isoforms can differentially coordinate membrane remodeling and cytoskeletal dynamics during endocytic progression.

Beyond their roles in endocytosis, our findings suggest that myosin-1C and myosin-1D may have distinct functions during mitosis in *Dictyostelium’s* semi-closed mitosis. Myosin-1D uniquely occupies the entire inner volume of the nuclear boundaries additionally colocalizing with spindle microtubules at later stages. This behavior implicates it to potentially being involved in nuclear envelope dynamics, chromatin organization and/or mitotic progression, possibly facilitated by the nuclear envelope fenestration. This contrasts with the spindle-restricted localization of myosin-1C throughout the entire division process, indicative of divergent mitotic roles among the myosins. Binding of myosin-1D tail constructs to isolated, membrane-enclosed nuclei suggest phosphoinositide interactions—likely with PI(4,5)P₂, the predominant nuclear envelope phosphoinositide in both *Dictyostelium* and mammals—as a potential mechanism for nuclear targeting. This is consistent with emerging evidence that phosphoinositide signaling at the nuclear envelope influences mitotic progression and nuclear integrity [102].

Additionally, differential microtubule-binding capacities among myosin-1 isoforms highlight their functional diversification during cell division. Our data support a model wherein specific phosphoinositide recognition, transient interactions with actin and microtubules, and possibly further contacts with chromatin and nuclear envelope components collectively contribute to myosin-1D’s mitotic localization. Although the precise molecular mechanisms underlying nuclear targeting remain to be elucidated, phosphoinositide recognition and potential interactions with nuclear components, appear to direct myosin-1D to this compartment, suggesting complementary mitotic roles. Despite the identification of myosin-1D’s nuclear association during mitosis, its functional contributions remain unclear. Its potential association with membranous nuclear structures, inner-nuclear distribution and partial colocalization with spindle microtubules and spindle poles suggests potential involvement in assisting cell division through roles in chromatin remodelling, similar to functions reported for NMI [48]. Alternatively, myosin-1D may participate in membrane trafficking or cytoskeletal reorganization processes associated to *Dictyostelium*’s semi-closed mitosis. Given myosin-1D’s diverse and dynamic subcellular localization—including its presence in the nucleus—it is also conceivable that the myosin transiently interacts with the Golgi apparatus. Such interactions might occur during vesicle trafficking or membrane remodelling, though these roles have yet to be explicitly demonstrated and may represent a secondary function. At this point, the specific nuclear role of myosin-1D during mitosis remains unassigned, highlighting the need for further investigation.

In summary, our data support a model in which phosphoinositide specificity, actin-binding affinity, and isoform expression converge to govern myosin-1 targeting and function. This type of regulation enables distinct myosin-1 isoforms to coordinate membrane remodeling and cytoskeletal dynamics with precise temporal and spatial control, ensuring efficient endocytosis and faithful mitotic progression. These findings advance our understanding of how motor proteins decode complex lipid and cytoskeletal cues to fulfil specialized cellular roles.

## Supplementary Information

Movie M1. Confocal time-lapse imaging showing localization of myosin-1C, myosin-1D, F-actin, CRAC-PH, and TAPP2-PH during micropinocytosis. Time is indicated in minutes and seconds. Scale bars, 5 μm.

Movie M2. Confocal time-lapse imaging showing localization of myosin-1C, myosin-1D, F-actin, and CRAC-PH during yeast particle phagocytosis. Time is indicated in minutes and seconds (min:sec). Scale bars, 5 μm.

Movie M3. Confocal time-lapse imaging showing localization of myosin-1C, myosin-1D, F-actin, CRAC-PH, and TAPP2-PH during micropinocytosis in the presence of the PIK3 inhibitor LY294002. Time is indicated in minutes and seconds (min:sec). White arrows indicate the macropinocytic events. Scale bars, 5 μm.

Movie M4. Confocal time-lapse imaging showing localization of myosin-1C, myosin-1D, F-actin, and CRAC-PH during yeast particle phagocytosis in the presence of the PIK3 inhibitor LY294002. Time is indicated in minutes and seconds (min:sec). White arrows indicate the phagocytic events. Scale bars, 5 μm.

Movie M5. Confocal time-lapse imaging showing the localization of myosin-1D during mitosis. Time is indicated in minutes and seconds (min:sec). Scale bars, 5 μm.

Supplementary Material 6.

Supplementary Material 7.

## Data Availability

No datasets were generated or analysed during the current study.
