# Supplementary material for: Long-tailed class I myosins rely on tail-mediated phosphoinositide recognition for specific membrane recruitment
Source: Cell Commun Signal. 2025 Dec 4;23:519. doi: 10.1186/s12964-025-02528-x (PMC12676778; doi:10.1186/s12964-025-02528-x)
Supplement: Supplementary file 6 — Supplementary Material 6. [file 12964_2025_2528_MOESM6_ESM.docx]

**SUPPLEMENTARY FIGURES**


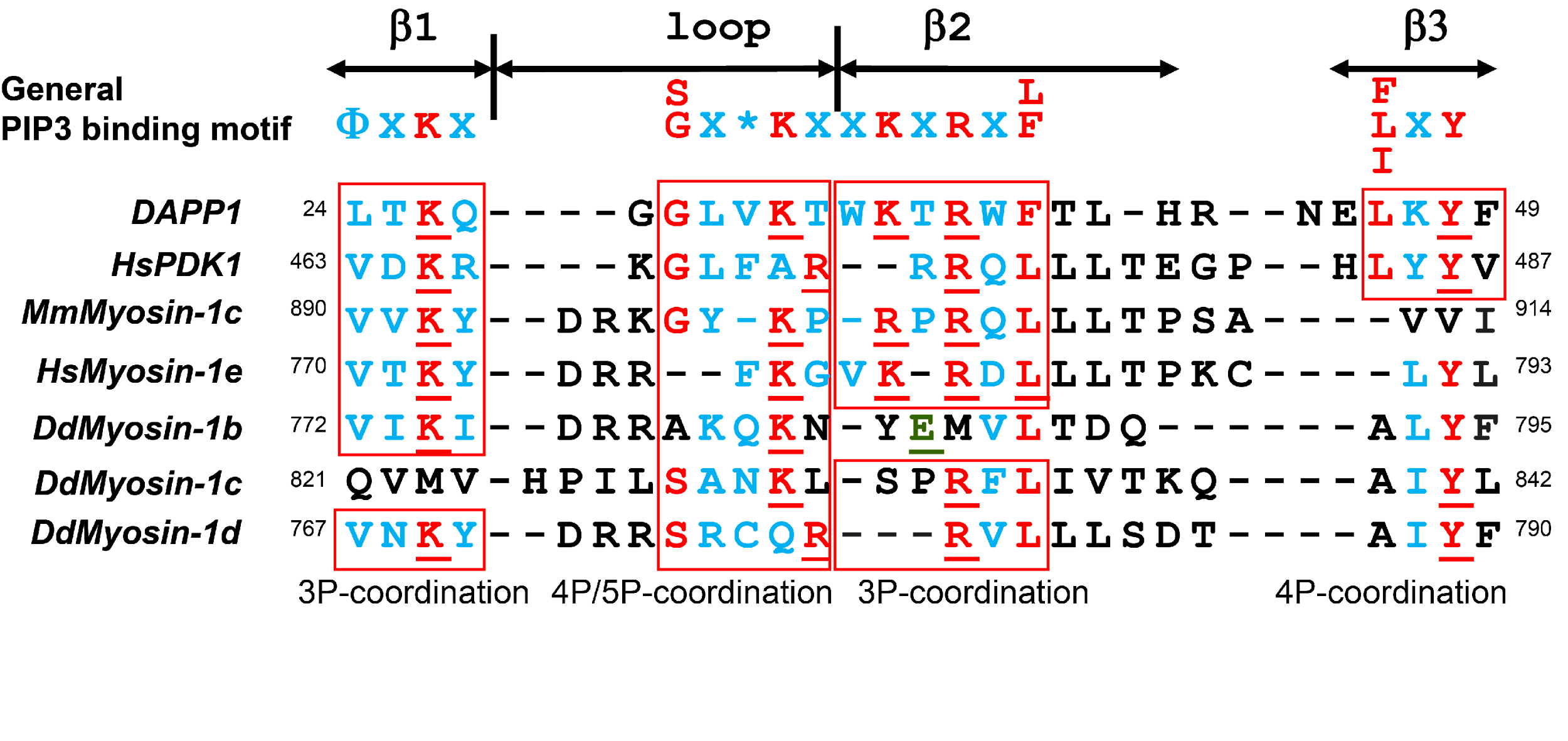


**Supplementary Fig. S1:** **Sequence alignment of PH domains of class-1 myosin tails and PIP3 high affinity binders.** Comparisons with conserved motifs that mediate PIP3 specificity reveal intrinsic differences in the β1, β2, β3 sheets and β1-β2 loop which potentially coordinate specific phosphate group interactions. The proteins, DAPP1 (dual adaptor of phosphotyrosine and 3-phosphoinositides) and PDK1 (phosphoinositide dependant kinase-1) bind to PIP3 with high specificity. The consensus sequence for PH domains that bind PIP3 specifically is shown on top. In the consensus sequence ‘Φ’ represents an amino acid with hydrophobic side chain; ‘X’ represents any amino acid; ‘*’ represents any number of amino acids (0 or more). Conserved amino acids are indicated in red and amino acid residues that are reported to be involved in direct binding to phosphate groups are underlined.

**
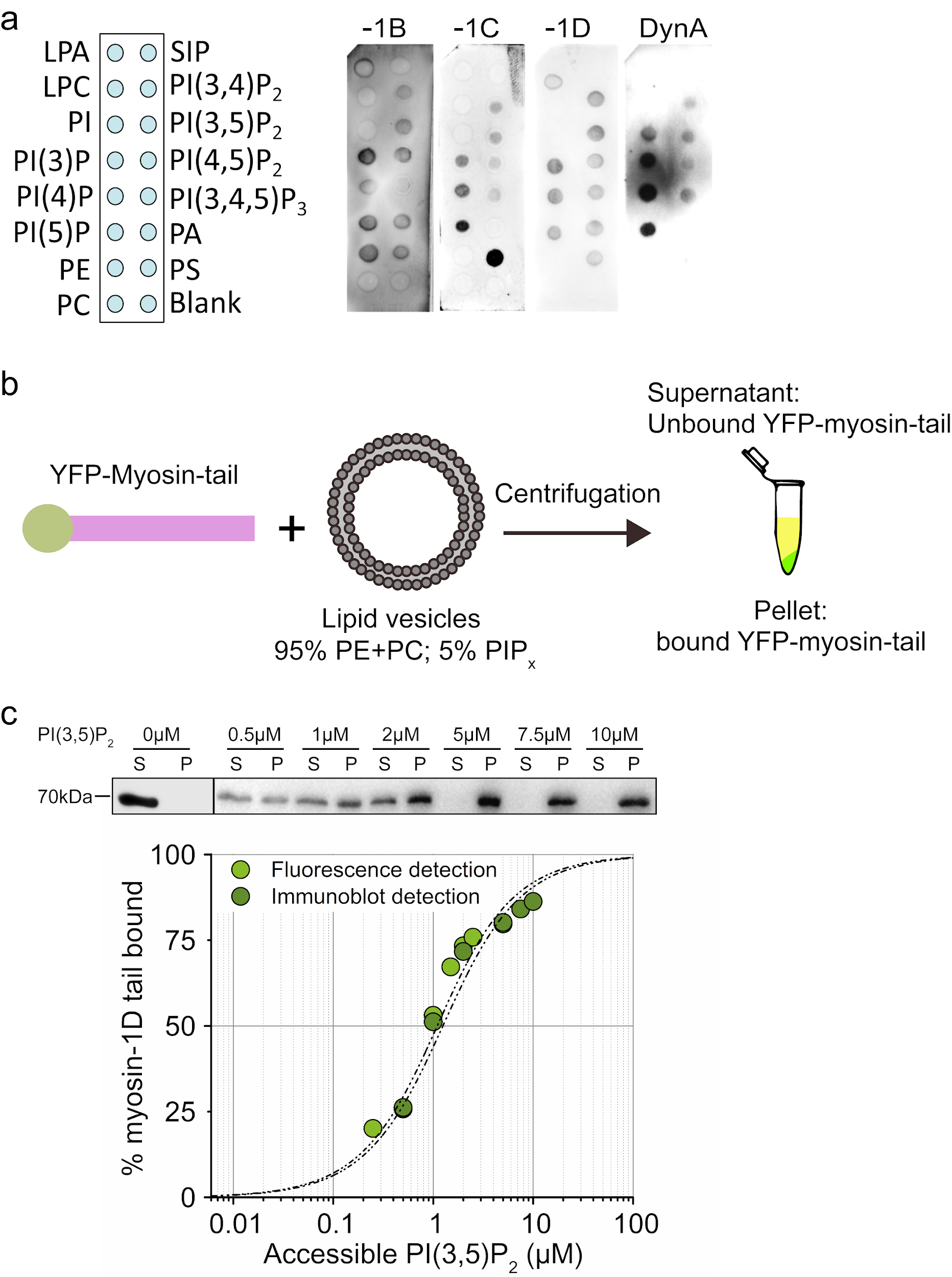
**

**Supplementary Fig. S2. Lipid dot blot assays and comparisons between fluorescence-based detection and immunoblot detection of myosin-1-PI(3,5)P_2_ interactions. (a)** Lipid dot-blot assay. Representative images of three or more independent experiments with purified tail domains of *Dd*myosin-1B, *Dd*myosin-1C, *Dd*myosin-1D and *Dd*dynamin-A as a positive control. PIP Strips contain the following lipids at 100 pmol per spot: Lysophosphatidic acid (LPA), Lysophosphocholine (LPC), Phosphatidylinositol (3) phosphate (PI(3)P), Phosphatidylinositol (4) phosphate (PI(4)P), Phosphatidylinositol (5) phosphate (PI(5)P), Phosphatidylethanolamine (PE), Phosphatidylcholine (PC), Sphingosine 1-Phosphate (S1P), Phosphatidylinositol (3,4) bisphosphate (PI(3,4)P_2_), Phosphatidylinositol (3,5) bisphosphate (PI(3,5)P_2_), Phosphatidylinositol (4,5) bisphosphate (PI(4,5)P_2_), Phosphatidylinositol (3,4,5) trisphosphate (PI(3,4,5)P_3_), Phosphatidic acid (PA), and Phosphatidylserine (PS). (**b**) Schematic representation of the experimental workflow to quantify the affinity of class-1 myosins to lipids. (**c**) Quantification of binding affinities of *D. discoideum* myosin-1 tails to anionic lipids. Representative blot for sedimentation titration of 0.5µM GST-myosin-1D tail with varying concentrations of PI(3,5)P_2_ with supernatant and pellet after centrifugation (**upper panel**). Tagged myosin tails were mixed with varying concentrations of PolyPIPosomes^TM^. The reaction was centrifuged and the amount of myosin tails present in the supernatant and pellet were quantified either by SDS-PAGE followed by western blotting probed with anti-GST antibody or by the use of fluorimeter as described in the Materials and Methods. Titration curves of lipid binding exemplary for myosin-1D tail to PI(3,5)P_2_ obtained by immunoblotting based vs fluorescence detection (**lower panel**).

**
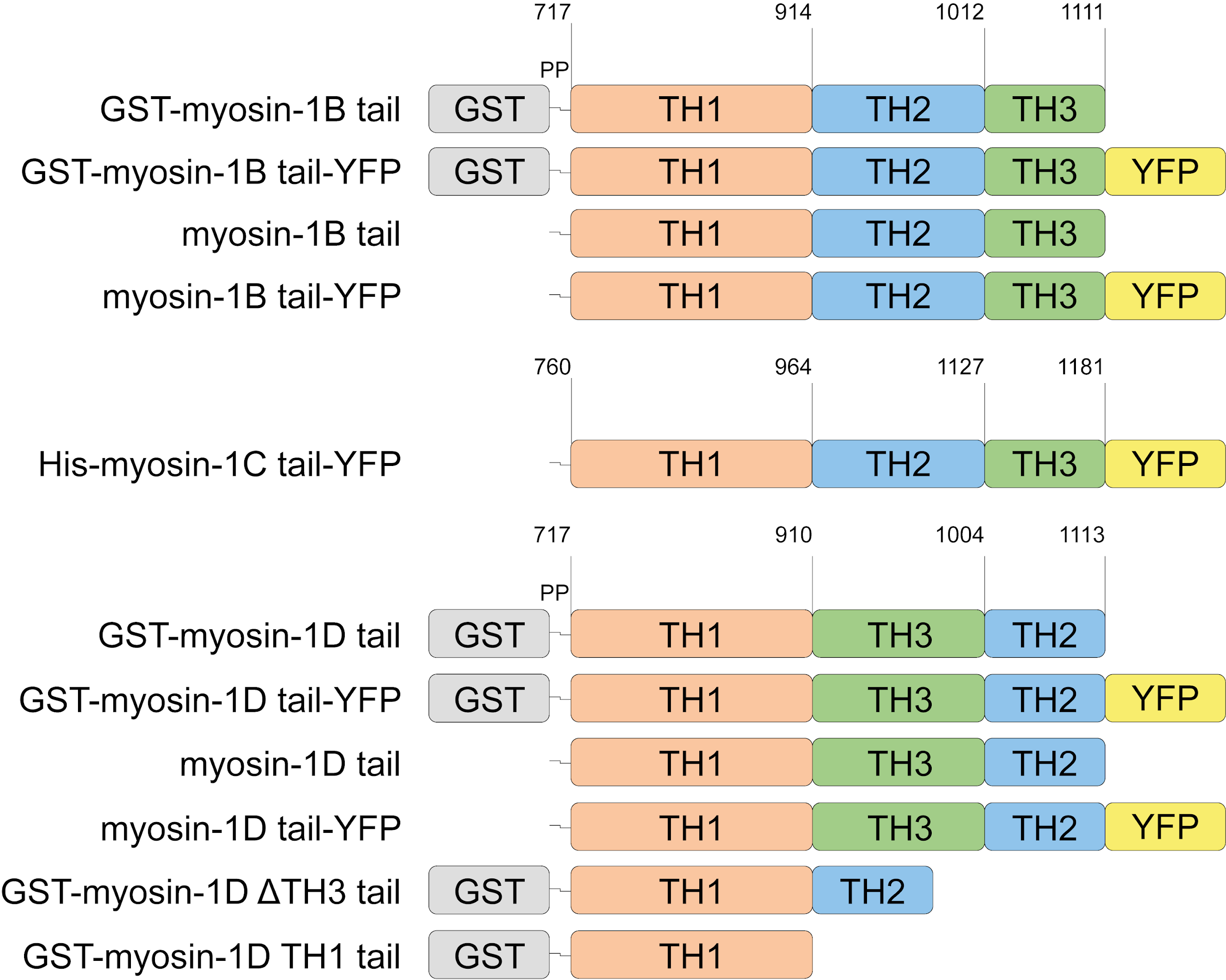
**

**Supplementary Fig. S3. Myosin-1 constructs used in the study**


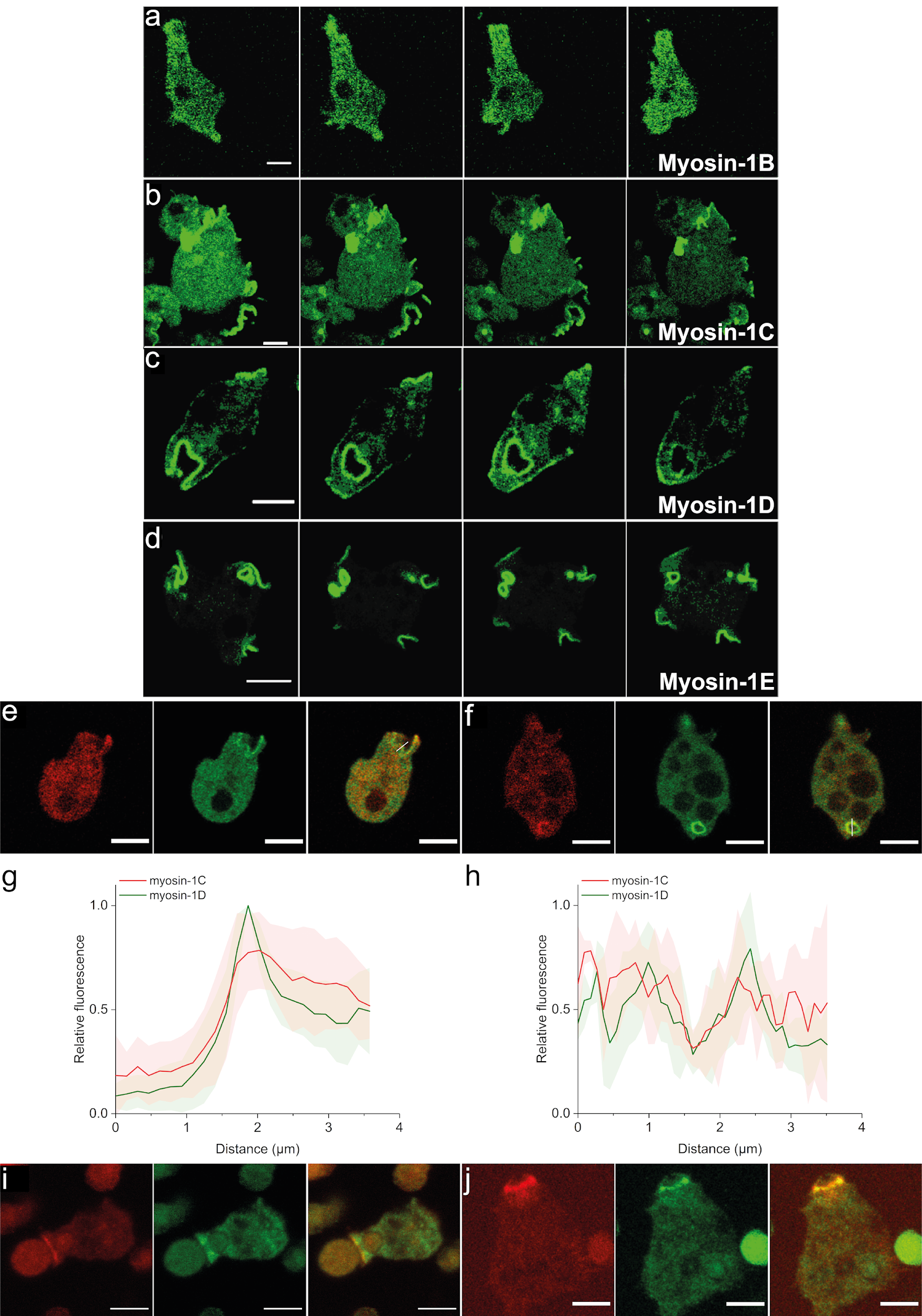


**Supplementary Fig. S4. Colocalization of myosin-1C and myosin-1D during macropinocytosis and phagocytosis.** (**a-d**) Localization of myosin-1B, myosin-1C, myosin-1D and myosin-1E in AX2 cells transfected with expression plasmids encoding YFP-tagged variants of the proteins. (**e,f**) Colocalization of myosin-1C and myosin-1D in macropinocytic AX2 cells after cotransfection with expression plasmids encoding YFP-tagged myosin-1D and RFP-tagged myosin-1C, respectively under starving conditions. (**g, h**) Fluorescence intensity profiles of RFP-myosin-1C and YFP-myosin-1D at the plasma membrane during macropinocytosis. (**i,j**) Colocalization of myosin-1C and myosin-1D in phagocytic AX2 cells after cotransfection with expression plasmids encoding YFP-tagged myosin-1D and RFP-tagged myosin-1C, respectively under starving conditions. Scale bar 5 µm.

**
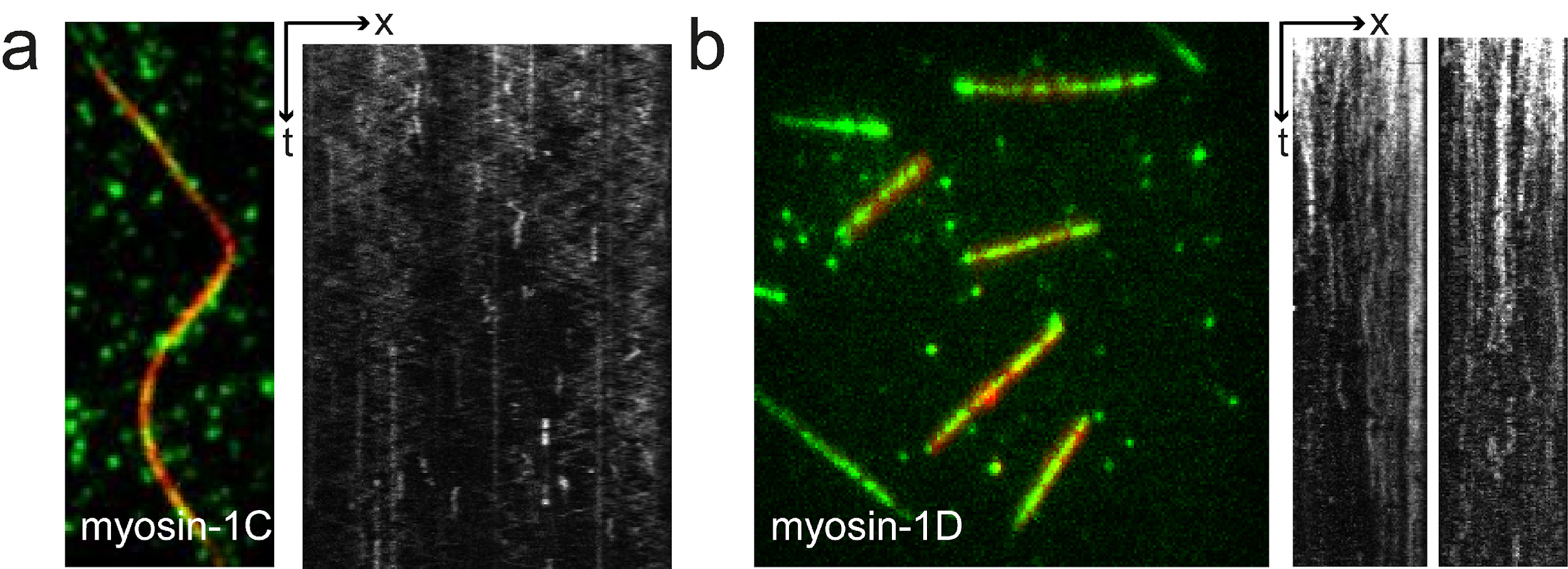
**

**Supplementary Fig. S5**. **Diffusion of myosin-1C and myosin-1D along microtubules.** (**a**) TIRF-microscopy image showing YFP-myosin-1C-tail (green) binding to and diffusing along surface-immobilized microtubules (red). Corresponding kymographs (right panel). (**b**) TIRF-microscopy image showing YFP-myosin-1D-tail (green) binding to and diffusing along surface-immobilized microtubules (red). Corresponding kymographs (right panel).


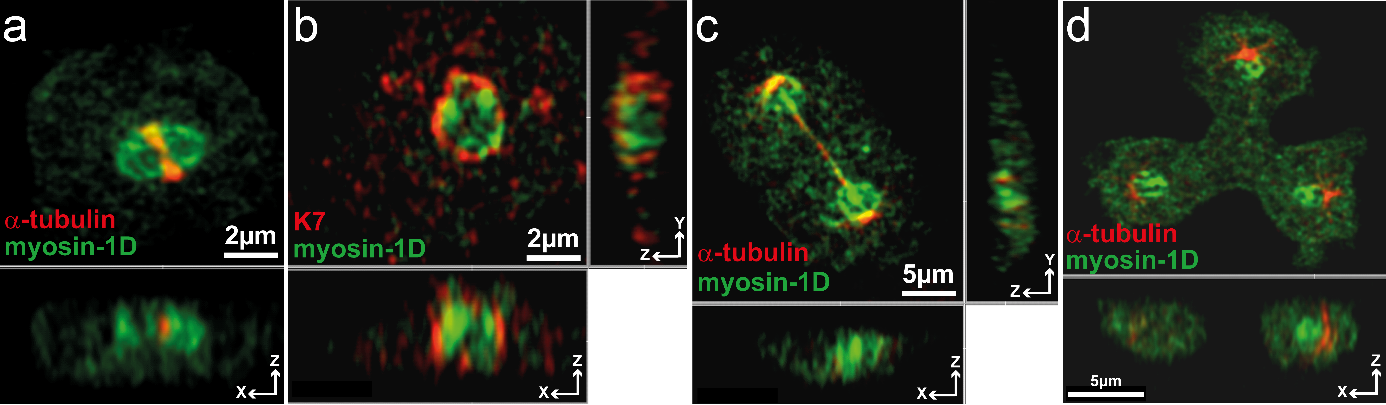


**Supplementary Fig. S6. Nuclear localization of myosin-1D during mitosis.** (**a**) Confocal micrograph and xz projection of an early metaphase cell expressing myosin-1D (green), stained for tubulin (red). (**b**) Confocal micrograph and xz/yz projections of a prometaphase/metaphase cell expressing myosin-1D (green) stained for K7 (red). (**c**) Confocal micrograph and projections of the xz/yz layers of a cell at telophase showing myosin-1D (green) localization at the nuclei and colocalizing with microtubules (red) at the spindle poles. (**d**) Confocal micrograph of a triple nucleated cell performing cytokinesis. Myosin-1D localizes with the nuclear masses.
